# Unraveling the MAX2 Protein Network in *Arabidopsis thaliana*: Identification of the Protein Phosphatase PAPP5 as a Novel MAX2 Interactor

**DOI:** 10.1074/mcp.RA119.001766

**Published:** 2021-01-07

**Authors:** Sylwia Struk, Carolien De Cuyper, Anse Jacobs, Lukas Braem, Alan Walton, Annick De Keyser, Stephen Depuydt, Lam Dai Vu, Ive De Smet, François-Didier Boyer, Dominique Eeckhout, Geert Persiau, Kris Gevaert, Geert De Jaeger, Sofie Goormachtig

**Affiliations:** 1 Department of Plant Biotechnology and Bioinformatics, Ghent University, Ghent, Belgium; 2 Center for Plant Systems Biology, VIB, Ghent, Belgium; 3 Department of Biochemistry, Ghent University, Ghent, Belgium; 4 Center for Medical Biotechnology, VIB, Ghent, Belgium; 5 Institut Jean-Pierre Bourgin, Institut National de la Recherche Agronomique (INRA), AgroParisTech, Centre National de la Recherche Scientifique (CNRS), Université Paris-Saclay, Versailles, France; 6 Institut de Chimie des Substances Naturelles, CNRS Unité Propre de Recherche 2301, Université Paris-Sud, Université Paris-Saclay, Gif-sur-Yvette, France

**Keywords:** MAX2, strigolactones, karrikins, affinity purification, phosphorylation, seed germination, AP, affinity purification, BiFC, Bimolecular Fluorescent Complementation, *BZS1*, *bzr1–1D Suppressor 1*, *CHS*, *CHALCONE SYNTHASE*, Co-IP, coimmunoprecipitation, COP9, CONSTITUTIVE PHOTOMORPHOGENIC 9, CSN, COP9 signalosome, D14, DWARF 14, D53, DWARF53, *DLK2*, *D14-LIKE2*, FDR, false discovery rate, GS, protein G/streptavidin-binding peptide, *htl-3*, *hyposensitive to light 3*, *HY5*, *ELONGATED HYPOCOTYL 5*, KAI2, KARRIKIN INSENSITIVE 2, KAR, karrikin, KL, KAI2 ligand, LFQ, label-free quantification, MAX2, MORE AXILLARY GROWTH 2, PAPP5, PHYTOCHROME ASSOCIATED PROTEIN PHOSPHATASE 5, PMSF, phenylmethylsulfonyl fluoride, *rac*-GR24, synthetic strigolactone analog, SCF, S-phase kinase-associated protein 1 (Skp1), Cullin-1/CDC53 and F-box protein, SL, strigolactone, SMAX1, SUPPRESSOR OF MAX2 1, SD, synthetic-defined, SMXL, SMAX1-LIKE, *STH7*, *SALT TOLERANCE HOMOLOG7*, TAP, tandem affinity purification, Y2H, yeast two-hybrid

## Abstract

The F-box protein MORE AXILLARY GROWTH 2 (MAX2) is a central component in the signaling cascade of strigolactones (SLs) as well as of the smoke-derived karrikins (KARs) and the so far unknown endogenous KAI2 ligand (KL). The two groups of molecules are involved in overlapping and unique developmental processes, and signal-specific outcomes are attributed to perception by the paralogous α/β-hydrolases DWARF14 (D14) for SL and KARRIKIN INSENSITIVE 2/HYPOSENSITIVE TO LIGHT (KAI2/HTL) for KAR/KL. In addition, depending on which receptor is activated, specific members of the SUPPRESSOR OF MAX2 1 (SMAX1)-LIKE (SMXL) family control KAR/KL and SL responses. As proteins that function in the same signal transduction pathway often occur in large protein complexes, we aimed at discovering new players of the MAX2, D14, and KAI2 protein network by tandem affinity purification in *Arabidopsis* cell cultures. When using MAX2 as a bait, various proteins were copurified, among which were general components of the Skp1-Cullin-F-box complex and members of the CONSTITUTIVE PHOTOMORPHOGENIC 9 signalosome. Here, we report the identification of a novel interactor of MAX2, a type 5 serine/threonine protein phosphatase, designated PHYTOCHROME-ASSOCIATED PROTEIN PHOSPHATASE 5 (PAPP5). Quantitative affinity purification pointed at PAPP5 as being more present in KAI2 rather than in D14 protein complexes. In agreement, mutant analysis suggests that *PAPP5* modulates KAR/KL-dependent seed germination under suboptimal conditions and seedling development. In addition, a phosphopeptide enrichment experiment revealed that PAPP5 might dephosphorylate MAX2 *in vivo* independently of the synthetic SL analog, *rac*-GR24. Together, by analyzing the protein complexes to which MAX2, D14, and KAI2 belong, we revealed a new MAX2 interactor, PAPP5, that might act through dephosphorylation of MAX2 to control mainly KAR/KL-related phenotypes and, hence, provide another link with the light pathway.

## Highlights

• TAP revealed PAPP5 as a novel interactor of MAX2.
• PAPP5 might act through dephosphorylation of MAX2.
• PAPP5 modulates KAI2-controlled responses in seeds and young seedlings.
• PAPP5 might be another link between KAR/KL signaling and light.

## Graphical Abstract

**Figure:**
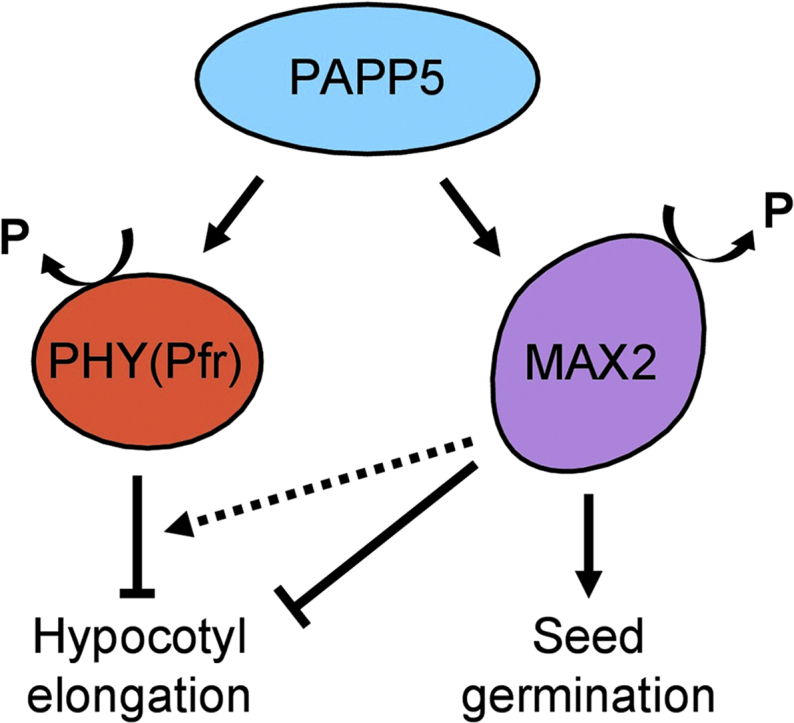

## In Brief

The F-box protein MAX2 is a central component in the signaling pathway of the phytohormones, strigolactones and smoke-derived karrikins. These molecules are perceived by the related receptors D14 and KAI2, respectively. Here, we used tandem and single affinity purifications to broaden the MAX2, KAI2, and D14 interaction network and identified PAPP5 as a novel player. PAPP5 might act *via* dephosphorylation of MAX2 to modulate KAI2-dependent seed germination and young seedling phenotypes.

F-box proteins represent one of the largest and most heterogeneous superfamilies in plants that modulate crucial processes from embryogenesis to seedling development, hormone signaling, and defense pathways (1). They confer substrate specificity of the S-phase kinase-associated protein 1 (Skp1), Cullin-1/CDC53, and F-box protein (SCF) E3 ubiquitin ligase complex that selects proteins for ubiquitination to mark them for proteolysis by the 26S proteasome (2, 3). All F-box proteins are characterized by an N-terminally conserved 40- to 50-amino acid F-box motif that interacts with Skp1 and a C-terminal target-binding domain (1).

One of the *Arabidopsis thaliana* F-box proteins, MORE AXILLARY GROWTH 2 (MAX2) is a central component of two distinct signaling pathways, one that perceives the plant hormones strigolactones (SLs) and one that senses the smoke-derived karrikins (KARs) (4, 5). SLs and KARs have been found to regulate different aspects of plant growth and development, and they also have some overlapping functions. SLs control shoot branching (6, 7), leaf senescence (8, 9), and secondary growth (10), whereas KARs activate seed germination under suboptimal conditions and regulate early seedling development (11, 12, 13). Both pathways have been described to influence root architecture (14, 15, 16, 17) and leaf growth (18) and to mediate stress responses in plants (19, 20, 21). Recently, MAX2 has also been shown to be involved in the regulation of callus formation, seed size, and seed yield (22).

Upon perception, plants need to activate specific signaling cascades to trigger a particular outcome. The SCF^MAX2^ specificity is dictated by interaction with the receptor proteins DWARF 14 (D14) that binds SLs (23, 24, 25, 26, 27) and its paralog KAI2, required for KAR responses (11, 28, 29, 30). As the *kai2* mutant displays developmental defects and KARs enhance germination and seedling photomorphogenesis of non-fire-prone species, KAI2 has been proposed to perceive also a thus far unknown, endogenous molecule, designated KAI2 ligand (KL) (31). Another level of discrimination between the two signaling cascades is provided by the SCF^MAX2^ degradation targets identified as SUPPRESSOR OF MAX2 1 (SMAX1)-LIKE (SMXL) proteins in *Arabidopsis* and their ortholog DWARF53 (D53) in *Oryza sativa* (rice) (32, 33, 34). Indeed, different SMXL family members are targeted for degradation depending on the activated signaling pathway and developmental steps. Members of the subclade 1, SMAX1 and SMXL2, are involved in KAR/KL-regulated growth responses (13, 33), whereas subclade 4 proteins, SMXL6, SMXL7, and SMXL8, are ubiquitinated and degraded in response to SLs (18, 35, 36). D53 of rice has been shown to group within the SMXL6, SMXL7, and SMXL8 phylogenetic clade (18). Recently, a common mechanism has been proposed for the regulation of hypocotyl responses, in which both receptors, D14 and KAI2, can mediate polyubiquitination and degradation of SMXL2 triggered by SL and KAR, respectively, providing evidence of cross talk between both signaling pathways (37).

By means of various biochemical and structural studies, D14 has been suggested to undergo a conformational change upon SL perception and to interact with the SCF^MAX2^ complex and the SMXL6, SMXL7, and SMXL8 proteins, whereafter the latter are targeted for ubiquitination and degradation (18, 35). The rice MAX2 ortholog DWARF3 (D3) has been found to possess a C-terminal α-helix that can adopt two conformational states, namely, one that enables D14 to recruit D53 in a SL-dependent manner and one that inhibits the enzymatic activity of the receptor (38). Central repressors of the SL signaling, SMXL6, SMXL7, and SMXL8, interact with TOPLESS and TOPLESS-RELATED proteins to control shoot branching, leaf elongation, and anthocyanin biosynthesis, mainly by inhibiting the activity of the BRANCHED 1 (BRC1), TCP DOMAIN PROTEIN 1 (TCP1), and PRODUCTION OF ANTHOCYANIN PIGMENT 1/2 (PAP1)/PAP2 transcription factors, respectively. Of interest, SMXL6 and SMXL7 can also act as transcription factors that regulate their own expression by direct binding to their promoters, hence creating a negative feedback loop for the SL pathway (39). Although the mode of action of the KAI2 signaling is less clear, the perception of KAR/KL or *rac*-GR24 by KAI2 has been found to cause ubiquitination after the degradation of SMAX1 by SCF^MAX2^. However, in contrast to the SL pathway, there is also evidence for a MAX2-independent degradation of SMAX1 (40).

The SCF^MAX2^ targets have been identified by means of a genetic approach (32, 33, 41), whereas biochemical approaches, including complex structural analysis, modeling, and dedicated protein–protein interactions have revealed many insights into the SL signaling complex (18, 23, 26, 35, 38). A complementary manner to comprehend the mode of action of a given signaling protein is the use of affinity purification (AP) to discover interaction partners and discern the protein complex structure to which the bait belongs (42). Although the direct downstream targets of both the MAX2-D14 and MAX2-KAI2 complexes have been characterized, many questions remain regarding the function and regulation of both pathways. Discovery of the complex-specific interaction partners might help to understand how these complexes orchestrate specific responses in plants and why both pathways are required for some phenotypes.

Here, we used tandem affinity purification (TAP) followed by mass spectrometry (MS) analysis in cell cultures to identify MAX2, D14, and KAI2-associated proteins to obtain insight into their function during SL and KAR signaling. In addition, we carried out a quantitative single-step AP to compare the D14 and KAI2 interactomes. As a result, a novel interactor was detected, PHYTOCHROME-ASSOCIATED PROTEIN PHOSPHATASE 5 (PAPP5), that seemingly acts preferentially with the KAI2 compared with the D14 pathway. Furthermore, we show that PAPP5 has a modulatory function in regulating numerous biological responses in seeds and young seedlings. In addition, analysis of MAX2 phosphorylation status revealed that PAPP5 might act through dephosphorylation of MAX2.

## Experimental Procedures

### Vector Construction

Transgenes encoding fusions with the protein G/streptavidin-binding peptide GS and GFP under control of the constitutive cauliflower tobacco mosaic virus *35S* promoter (CaMV-35S) were cloned by means of Gateway recombination (Thermo Fisher Scientific). The ORFs of MAX2, MAX2ΔFBOX, D14, KAI2, PAPP5 (splice variant AT2G42810.1), and the MAX2 promoter region (*pMAX2*, 2031 bp) were amplified from *Arabidopsis* cDNA with iProof High-Fidelity DNA Polymerase (Bio-Rad) and Gateway-specific primers (see supplemental Table S1). For the construction of the modified version of MAX2 (hereafter designated MAX2ΔFBOX), the first 159 nucleotides were deleted. The attB site-flanked PCR product was cloned into pDONR207 (ORF) or pDONRP4-P1R (*pMAX2*) with the BP Clonase II enzyme mix (Invitrogen). The entry vectors were subsequently cloned with the LR Clonase II Plus enzyme mix (Invitrogen) into the destination vectors pKNTAP for N-terminal GS fusions (42) or into pK7m34GW for the N- and C-terminal GFP fusions. The obtained expression clones were transformed to the *Agrobacterium tumefaciens* strain C58C1Rif^R^ (pMP90) by electroporation.

### Cell Culture Transformation

*A. thaliana* (L.) Heynh. (accession Landsberg *erecta*) cell suspension cultures (PSB-D, Arabidopsis Biological Research Center, clone no. CCL84840) were transformed through cocultivation with *A. tumefaciens* containing either the GS or GFP protein fusions (43). After transformation, the liquid MSMO medium (4.43 g/l Murashige and Skoog basal salts with minimal organics [Sigma-Aldrich], 30 g/l sucrose, 0.5 mg/l α-naphtaleneacetic acid, 0.05 mg/l kinetin, pH 5.7) supplemented with a mixture of three antibiotics (25 μg/ml kanamycin, 500 μg/ml carbenicillin, and 500 μg/ml vancomycin) was used for the selection of transgenic cell cultures. Transgene expression levels were analyzed 3 weeks after cocultivation by Western blot as described below. Cultures expressing the bait protein were maintained in fresh MSMO medium at 25 °C under continuous dark (24 h, TAP) or light/dark (16 h/8 h, GFP-trap) conditions by gentle agitation (130 rpm) and, subsequently, upscaled for TAP or GFP-trap analysis.

### Tandem Affinity Purification

Cell culture material was harvested after 5 h of treatment with the synthetic SL analog *rac*-GR24 (1 μM) or 0.01% (v/v) acetone (mock). TAP experiments (42) were done with some modifications. Briefly, proteins were extracted with the extraction buffer (25 mM Tris-HCl, pH 7.6, 15 mM MgCl_2_, 150 mM NaCl, 15 mM *p*-nitrophenyl phosphate, 60 mM β-glycerophosphate, 0.1% [v/v] NP-40, 0.1 mM Na_3_VO_4_, 1 mM NaF, 1 mM phenylmethylsulfonyl fluoride [PMSF], 1 μM E64, EDTA-free Ultra complete tablet [1/10 ml; Roche Diagnostics], 0.1% [v/v] benzonase, and 5% [v/v] ethylene glycol). After concentration determination by the Bradford assay (Bio-Rad), 25 mg of total protein extract was incubated with IgG-Sepharose 6 Fast Flow beads (GE Healthcare), pre-equilibrated with extraction buffer, for 1 h at 4 °C. After the washing step on the Mobicol column with wash buffer (10 mM Tris-HCl, pH 7.6, 150 mM NaCl, 0.1% [v/v] NP-40, 0.5 mM EDTA, 1 μM E64, 1 mM PMSF, and 5% [v/v] ethylene glycol), the beads were incubated with AcTEV protease (2 × 100 units; Invitrogen) for 1 h at 16 °C. The IgG elute was incubated with streptavidin beads (GE Healthcare), pre-equilibrated with wash buffer, for 1 h at 4 °C. The protein complexes were eluted by addition of 1 ml streptavidin elution buffer (20 mM desthiobiotin in wash buffer), and proteins were concentrated by TCA precipitation (final concentration 25%) at 4 °C overnight. The protein pellet was resolved in 30 μl of 1× NuPAGE sample buffer (Invitrogen). Proteins were separated on 4% to 12% gradient NuPAGE Bis-Tris gels (Invitrogen). After the proteins had been separated, they were visualized with colloidal Coomassie Brilliant Blue G-250 (Sigma-Aldrich) and in-gel digested with trypsin (MS Gold; Promega) for 3.5 h at 37 °C as described (42). In total for each condition (mock and 1 μM *rac*-GR24), two replicates were done for the cell cultures PSB-D expressing *35S::GS-MAX2*, *35S::GS-MAX2ΔFBOX, 35S::GS-D14*, and *35S::GS-KAI2*.

### LC-MS/MS Analysis

The peptide mixtures obtained after trypsin digest of the TAP samples were analyzed by LC-MS/MS as described (42, 44) with a tandem UltiMate 3000 RSLCnano system (Thermo Fisher Scientific) in-line connected to an LTQ Orbitrap Velos mass spectrometer (Thermo Fisher Scientific) through a Pneu-Nimbus dual-column source (Phoenix S&T). The peptides were first loaded on a trapping column (made in-house, 100 μm internal diameter [ID] × 20 mm length, 5-μm beads C18 Reprosil-HD [Dr Maisch]) and then eluted and bound onto a reverse-phase analytical column (made in-house, 75 μm ID × 150 mm length, 5-μm beads C18 Reprosil-HD [Dr Maisch]). The peptides were solubilized in 20 μl loading solvent (0.1% [v/v] TFA in 98/2 water/acetonitrile [v/v]), of which 5 μl was loaded and separated with a linear gradient from 98% of solvent A (0.1% [v/v] formic acid in water) to 55% of solvent B (0.1% [v/v] formic acid in 20/80 [v/v] water/acetonitrile) in 30 min at a flow rate of 300 nl/min, and followed by a 5-min wash reaching 99% of solvent B. The mass spectrometer was operated in data-dependent, positive ionization mode, automatically switching between MS and MS/MS acquisition for the 10 most abundant peaks in a given MS spectrum. In the LTQ-Orbitrap Velos, full-scan MS spectra were acquired in the Orbitrap at a target value of 1E6 with a resolution of 60,000. The 10 most intense ions were isolated for fragmentation in the linear ion trap, with a dynamic exclusion of 40 s. Peptides were fragmented after filling the ion trap at a target value of 1E4 ion counts. The background polydimethylcyclicsiloxane ion at 445.120025 Da was used for internal calibration (lock mass).

### Single-Step Affinity Purification

The *Arabidopsis* cell cultures (PSB-D) overexpressing the *35S::D14-GFP* and *35S::KAI2-GFP* constructs were treated for 15 min with 1 μM *rac*-GR24. After harvest, the protein material was extracted and 25 mg of the total protein input was used to purify protein complexes with the anti-GFP agarose beads GFP-Trap-A (Chromotek) according to the manufacturer's instructions with some modifications. Briefly, approximately 2 g of plant material was ground with pestle and mortar in liquid nitrogen. The resulting powder was homogenized in the lysis buffer (10 mM Tris-HCl, pH 7.5, 150 mM NaCl, 0.5 mM EDTA, 0.5% [v/v] NP-40, 1 EDTA-free Ultra complete tablet [1/10 ml; Roche Diagnostics]), mixed for 1 min with a T 25 digital Ultra-Turrax homogenizer (VWR International), and incubated on ice for 20 min. The protein extract was cleared by centrifugation at 20,000*g* at 4 °C for 20 min, and the NP-40 in the protein extract was diluted to 0.2% (v/v) by addition of a dilution buffer (10 mM Tris-HCl, pH 7.5, 150 mM NaCl, 0.5 mM EDTA, 1 EDTA-free Ultra complete tablet [1/10 ml; Roche Diagnostics]). The mixture was incubated with 50 μl of anti-GFP agarose beads, pre-equilibrated with the dilution buffer, for 2 h at 4 °C with rotation. The proteins were eluted from the beads by adding twice 50 μl 100% (v/v) formic acid. The protein eluate was frozen and vacuum dried. The pellet was dissolved in 200 mM triethylammonium bicarbonate and digested with trypsin (enzyme-to-substrate ratio of 1:100 [w:w]) overnight at 37 °C (MS Gold; Promega).

### Phosphopeptide Enrichment

*Arabidopsis* seedlings expressing *35S::GS-MAX2* (accession Columbia-0) and *35S::GS-MAX2* (*papp5-1*) were grown for 6 days in liquid half-strength Murashige and Skoog medium with 1% (w/v) sucrose on a rotating platform (80–90 rpm) at 21 °C under a 16-h light/8-h dark cycle. The protein extracts were prepared by grinding 9 g (3 × 3 g for biological replicates) of seedlings in liquid nitrogen and supplemented with extraction buffer (25 mM Tris-HCl, pH 7.6, 15 mM MgCl_2_, 150 mM NaCl, 15 mM *p*-nitrophenyl phosphate, 60 mM β-glycerophosphate, 0.1% [v/v] NP-40, 0.1 mM Na_3_VO_4_, 1 mM NaF, 1 mM PMSF, 1 μM E64, EDTA-free Ultra complete tablet [1/10 ml; Roche Diagnostics], 0.1% [v/v] benzonase, 5% [v/v] ethylene glycol, and 1 Phosphatase Inhibitor Cocktail Tablet PhosSTOP [Roche Diagnostics]). Protein extracts were sonicated three times for 30 s with a 30-s pause on ice in between. The extracts were centrifuged twice at 14,000*g* at 4 °C for 15 min, and after protein concentration determination by the Bradford assay, 25 mg of protein input was added to 50 μl of magnetic IgG beads, equilibrated with the extraction buffer, followed by incubation for 45 min on a rotating wheel at 4 °C. The beads were washed three times with the extraction buffer, once with the extraction buffer without detergent, and additionally with NH_4_HCO_3_. Next, NH_4_HCO_3_ was removed and the beads were resuspended in 50 μl of NH_4_HCO_3_ and 1 μg (4 μl 0.25 μg/μl) Trypsin/Lys-C Mix (Promega) was added for 3 h before digestion. The beads were removed on a magnetic separator, and the supernatant was transferred to a new 1.5-ml protein low bind Eppendorf and 0.5 μg (2 μl 0.25 μg/μl) Trypsin/Lys-C Mix was added and incubated overnight.

After the single-step purification, the phosphoenrichment experiment was performed as described (45, 46, 47). In short, peptide mixtures were incubated with 1 mg MagReSyn Ti-IMAC microspheres (ReSyn Biosciences) for 30 min at room temperature with continuous mixing. The microspheres were washed once with 60% (v/v) acetonitrile, 1% (v/v) TFA, and 200 mM NaCl and twice with 60% (v/v) acetonitrile and 1% (v/v) TFA. The bound phosphopeptides were eluted with three volumes (80 μl) of elution buffer (40% [v/v] acetonitrile, 5% [v/v] NH_4_OH). Samples were acidified to pH 3 with 100% (v/v) formic acid. Prior to MS analysis, the samples were vacuum dried and redissolved in 50 μl of 2% (v/v) acetonitrile and 0.1% (v/v) TFA.

### LC-MS/MS Analysis

The peptide mixtures that resulted from AP-MS and phosphopeptide enrichment were analyzed by an LC-MS/MS system on an Ultimate 3000 RSLC nano LC (Thermo Fisher Scientific) in-line connected to a Q Exactive mass spectrometer (Thermo Fisher Scientific). The peptides were first loaded on a trapping column (made in-house, 100 μm ID × 20 mm, 5-μm beads C18 Reprosil-HD, [Dr Maisch]) with loading solvent (0.1% [v/v] TFA in water/acetonitrile, 2/98 [v/v]). After 4 min, a valve switch put the loading column in line with the analytical pump and the peptides were separated on a 200-cm μPAC column (C18-endcapped functionality, 300-μm-wide channels, 5-μm porous-shell pillars, interpillar distance of 2.5 μm, and a depth of 20 μm; Pharmafluidics), kept at a constant temperature of 50 °C. Peptides were separated with a nonlinear gradient from 99% solvent A’ (0.1% [v/v] formic acid in water) to 10% solvent B′ (0.1% [v/v] formic acid in water/acetonitrile, 20/80 [v/v]) in 5 min, further increasing to 30% solvent B’ in 95 min followed by an increase to 50% solvent B’ in 25 min before ultimately reaching 99% solvent B’ in another 1 min. The column was then washed at 99% solvent B’ for 5 min and equilibrated for 15 min with 98% solvent A’. The mass spectrometer was operated in data-dependent, positive ionization mode, automatically switching between MS and MS/MS acquisition for the five most abundant peaks in a given MS spectrum. The source voltage was 2.9 kV, and the capillary temperature was 275 °C. One MS1 scan (*m/z* 400−2000, AGC target 3 × 106 ions, maximum ion injection time 80 ms), acquired at a resolution of 70,000 (at 200 *m/z*), was followed by up to five tandem MS scans (resolution 17,500 at 200 *m/z*) of the most intense ions fulfilling predefined selection criteria (AGC target 5 × 10^4^ ions, maximum ion injection time 80 ms, isolation window 2 D, fixed first mass 140 *m/z*, spectrum data type: centroid, underfill ratio 2%, intensity threshold 1.3 x E4, exclusion of unassigned, 1, 5–8, >8 positively charged precursors, peptide match preferred, exclude isotopes on, dynamic exclusion time 12 s). The HCD collision energy was set to 25% Normalized Collision Energy and the polydimethylcyclosiloxane background ion at 445.120025 Da was used for internal calibration (lock mass).

### MS/MS Data Processing and Analysis

For TAP samples, the raw files were processed with the Mascot Distiller (version 2.3.2.0 and 2.4.3.1) as described (42). The data were searched against the Arabidopsis Information Resource TAIR10_pep_20101214 database (version April, 2014, 35,386 entries). The searches were carried out with the Mascot search engine (version 2.3.0.1 and 2.4.1). Fixed modifications were set to carbamidomethylation of cysteines, and variable modifications were set to oxidation of methionines and methylation of aspartic acid and glutamic acid. Trypsin\P was selected as the enzyme setting. Cleavage was allowed when arginine or lysine was followed by proline with one missed cleavage permitted. Peptide and Fragment Mass Tolerances were set to 10 ppm and 0.5 Da, respectively. Significance threshold and ions score threshold were set to 0.01. Only identifications with at least two matched high-confidence peptides, of which at least one was unique to the protein, were retained. Background proteins were filtered out based on the occurrence frequency of the copurified proteins in a large dataset containing 543 TAP experiments with 115 different baits both in *Arabidopsis* cell cultures and seedlings and including proteins copurified with the negative control GSrhino/GS tag alone (42). MS/MS data processing and analysis for AP samples were performed exactly as detailed (44). All raw files were processed with the MaxQuant software (version 1.4.1.2) (48). The derived data were searched with the built-in Andromeda search engine against the *A. thaliana* TAIR10_pep_20101214 database (version April, 2014, 35,386 entries). Carbamidomethylation of cysteines was selected as the fixed modification, whereas oxidation and acetylation (protein N-term) were set as variable modifications. Trypsin\P was selected as the enzyme setting. Cleavage was allowed when arginine or lysine was followed by proline with two missed cleavages permitted. Matching between runs was enabled with a matching window time of 30 s. Label-free quantification (LFQ) of proteins was selected by means of the MaxLFQ algorithm integrated into MaxQuant. With the minimum ratio count set to 1, the FastLFQ option was enabled, LFQ minimum number of neighbors was set to 3, and the LFQ average number of neighbors to 6, as per default. Proteins identified with at least one unique peptide were retained. The false discovery rate (FDR) for peptide and protein identifications was set to 1%, and the minimum peptide length was set to seven amino acids. Detailed MaxQuant search parameters can be found in supplemental Excel Sheet 1. After MS data processing, LFQ values from the MaxQuant output file were analyzed with the Perseus software (version 1.5.3.2) as described (44). A two-sided Student’s *t* test was carried out in combination with a permutation-based correction for multiple hypothesis testing (FDR = 0.05) and a threshold value at S0 = 0.1. The mass spectrometry proteomics data have been deposited to the ProteomeXchange Consortium *via* the PRIDE (49) partner repository with the dataset identifier PXD015657.

### Homology Modeling and Structures

The predicted crystal structure of AtMAX2 was modeled *via* the SWISS-MODEL webserver (50) (https://swissmodel.expasy.org/interactive) with the AtMAX2 protein sequence and the rice D3 crystal structure as a template (26). Protein structures and models were visualized with PyMOL (https://pymol.org/2/).

### Western Blot Analysis

*Arabidopsis* cell cultures expressing *35S::GS-PAPP5* were treated with 1 μM *rac*-GR24 or 0.01% (v/v) acetone (mock). Cell material was harvested before and at six time points after treatment. For the expression analysis of the bait protein, the material was harvested from cell cultures 3 days after cocultivation or from 6-day-old seedlings grown in liquid half-strength Murashige and Skoog medium with 1% (w/v) sucrose on a rotating platform (80–90 rpm) at 21 °C under a 16-h light/8-h dark cycle. Proteins were extracted with the extraction buffer (25 mM Tris-HCl, pH 7.6, 15 mM MgCl_2_, 150 mM NaCl, 15 mM *p*-nitrophenyl phosphate, 60 mM β-glycerophosphate, 0.1% [v/v] NP-40, 0.1 mM Na_3_VO_4_, 1 mM NaF, 1 mM PMSF, 1 μM E64, EDTA-free Ultra complete tablet [1/10 ml; Roche Diagnostics], 0.1% [v/v] benzonase, and 5% [v/v] ethylene glycol). The protein concentrations were determined by the Bradford assay (Bio-Rad). Protein extracts (60 μg) were separated by SDS-PAGE (12% Mini-PROTEANTGX precast gels, Bio-Rad) and blotted on a polyvinylidene fluoride membrane (Trans-Blot TurboTM Mini PVDF Transfer, Bio-Rad) according to the manufacturer’s instructions. Differences in TAP fusion and GFP-tagged protein levels were detected by Western blotting with peroxidase-anti-peroxidase antibody against GS tag (1:2500, Sigma-Aldrich) or anti-GFP-horseradish-peroxidase-conjugated antibody (1:2000, MACS Miltenyi Biotec), respectively. The signals were spotted by means of chemiluminescent substrates from the Western Lightning Plus Enhanced Chemiluminescence kit (PerkinElmer) and X-ray films (Amersham Hyperfilm ECL; GE Healthcare). The Precision Plus Protein Dual Color Standards (Bio-Rad) was used as protein size marker.

### Subcellular Localization Analysis

About 4- to 5-week-old *Nicotiana benthamiana* plants were used for transient expression of the constructs *35S::GFP-PAPP5* and *pMAX2::GFP-MAX2* by *A. tumefaciens* (C58C1 strain)-mediated transformation of lower epidermal leaf cells as described (51) by means of a modified infiltration buffer (10 mM MgCl_2_ [Merck], 10 mM MES, pH 5.7 [Duchefa], 100 μM acetosyringone [Sigma-Aldrich]). An *Agrobacterium* strain harboring a P19 viral suppressor of gene silencing was coinfiltrated to boost protein expression (52). All *Agrobacterium* strains were grown for 2 days and centrifuged (3220*g*), and the pellet was resuspended in the infiltration buffer, mixed in a 1:1 ratio with other strains (final absorbance = 1) and injected. After 72 h, expression of the constructs was visualized with a Zeiss LSM 710 confocal laser scanning microscope with a 20× objective (Plan-Apochromat, numerical aperture 1.0). GFP fluorescence was monitored by a 498- to 600-m emission and a 488-nm excitation line of an argon laser.

### Bimolecular Fluorescence Complementation

Expression clones containing the N- and C-terminal parts of GFP (nGFP and cGFP, respectively) either as N- or C-terminal fusions under the control of the CaMV-35S promoter were generated by double Gateway recombination with the pK7m24GW2 or pK7m34GW vector (51), respectively. The constructs were coexpressed in *N. benthamiana* by *Agrobacterium*-mediated transient transformation, and the interactions were scored by confocal microscopy, essentially as described above for the subcellular localization. Each interacting pair was assayed in three repeated experiments. Appropriate negative controls were carried out for all combinations.

### Coimmunoprecipitation Assay

The ORFs of candidate genes were cloned with GFP and 3× hemagglutinin (3HA) at their C-terminal part under the control of the CaMV-35S promoter in the pK7m34GW vector by means of Gateway cloning (51). The expression clones were subsequently coexpressed in *N. benthamiana* by *Agrobacterium*-mediated transient transformation as described above. To assess the effect of the SL analog on the interactions, 3 days after infiltration leaves were injected with 10 μM *rac*-GR24 or 0.1% (v/v) acetone (mock) for 3 h. The infiltrated *N. benthamiana* leaves (2 g) were ground in liquid nitrogen, and the proteins were extracted with the extraction buffer (50 mM Tris-HCl, pH 7.5, 150 mM NaCl, 10% [v/v] glycerol, 10 mM EDTA, 1 mM sodium molybdate, 1 mM NaF, 10 mM DDT, 0.5% [w/v] polyvinylpolypyrrolidone, 1% [v/v] protease inhibitor cocktail [Sigma-Aldrich; 1 tablet per 10 ml extraction buffer], 1% [v/v] NP-40). The extracts were centrifuged at 18,000*g* at 4 °C for 30 min, and the supernatant was passed through a 40-μm cell filter. The protein concentrations were determined by the Bradford assay, and extracts were diluted with the extraction buffer to a concentration of 2 mg/ml. A 1.5-ml extract was incubated with 20 μl anti-GFP agarose beads GFP-Trap-A (Chromotek) at 4 °C for 2 h. After incubation, the beads were washed three times with 1 ml of wash buffer (20 mM Tris-HCl, pH 7.5, 150 mM NaCl, 0.5% [v/v] NP-40). The washed beads were mixed with 40 μl Novex 2×Tris-Glycine SDS sample buffer (Thermo Fisher Scientific) and boiled for 10 min at 95 °C. Samples were separated by 12% SDS-PAGE and analyzed with anti-GFP-horseradish peroxidase–conjugated (1:2000, MACS Miltenyi Biotec) or with anti-hemagglutinin-horseradish peroxidase–conjugated (1:10,000, Abcam) antibody.

### Yeast Two-Hybrid

Expression clones for yeast two-hybrid (Y2H) were generated by LR Gateway recombination between the respective entry clones and pGILDA (bait) or pB42AD (prey). Y2H analysis was performed as described (53), in three independent repeats. The *Saccharomyces cerevisiae* EGY48 strain was cotransformed with the bait and prey by the PEG/lithium acetate method (54). Transformed yeasts were selected on Synthetic-Defined media supplemented with galactose and raffinose (SD Gal/Raf) and lacking Uracil (URA), Tryptophan (Trp), and Histidine (His) (Clontech). For each bait–prey combination, three individual colonies were grown overnight in liquid medium at 30 °C and 10- and 100-fold dilutions were dropped on control media (SD Gal/Raf-Ura-Trp-His) and selective media supplemented additionally with 5-bromo-4-chloro-3-indolyl-β-d-galactopyranoside (X-Gal, Duchefa). To test the influence of the SL analog on the interactions, 10 μM *rac*-GR24 or 0.1% (v/v) acetone (control) was added to the medium.

### Plant Material and Growth Conditions

Seeds of *A. thaliana* (L.) Heynh. (accession Columbia-0 [Col-0]) plants were surface sterilized by consecutive treatments of 70% (v/v) ethanol with 0.05% (w/v) SDS, washed with 95% (v/v) ethanol, sown on half-strength Murashige and Skoog medium with 1% (w/v) sucrose (for root phenotyping) or without (for hypocotyl analysis), supplemented with 0.01% (v/v) acetone (mock) or with 1 μM *rac*-GR24 and 0.007% (v/v) methanol (mock) or 1 μM KAR2 and stratified for 2 days at 4 °C. All the tested mutants are in Col-0 background. The *max2-1* (55), *htl-3* (56), *d14-1* (11), and *papp5-1* (57) have been described previously. The *papp5-3* (WiscDsLox368D06) mutant was purchased (Salk Institute for Biological Sciences) and the homozygotes were identified by genotyping.

### Shoot Branching

Rosette branches (shoots >1 cm) were counted of 6-week-old *Arabidopsis* plants grown in soil under a standard 16-h/8-h light/dark cycle (22 °C/18 °C) in controlled-environment rooms with light provided by white fluorescent tubes.

### Lateral Root Assay

After stratification, seedlings were grown vertically for 9 days in a 16-h/8-h light/dark cycle at 21 °C. The lateral root primordia were counted under a light microscope (S4E, Leica Microsystems). Plates were scanned, and the main root lengths were measured with the ImageJ 1.41 software (https://imagej.nih.gov/ij/) and a digitizer tablet (Wacom).

### Seedling Photomorphogenesis Assay

After stratification, seeds were exposed to white light for 3 h at 21 °C, returned to darkness for 21 h at 21 °C, and then exposed to continuous red light for 4 days at 21 °C. After scanning of the plates, the hypocotyls were measured with ImageJ 1.41 software.

### Thermoinduced Seed Dormancy Assay

The thermoinduced seed dormancy assay was performed similarly as described (58). Before utilization for the assay, harvested seeds were stored under ambient conditions for a minimum of 4 weeks. Seeds were distributed in a 96-well plate containing 100 mM Hepes buffer and 0.01% (v/v) PPM (preservative for plant tissue cultures; Plant Cell Technologies) supplemented with either 10 μM *rac*-GR24 or 0.1% (v/v) acetonitrile (mock). Plates were incubated for 4 to 6 days (for details, see figure legends) at 24 °C in the dark or at 21 °C under continuous light for the control. Germination was indicated by emergence of the radicle tip through the endosperm.

### RNA Extraction and Quantitative RT-PCR

RNA was extracted from wild-type *Arabidopsis* cell cultures treated with 0.01% (v/v) acetone (mock) or with 1 μM *rac*-GR24 for 1, 5, 10, or 15 h, from 4-day-old *Arabidopsis* seedlings grown under continuous red light on half-strength Murashige and Skoog medium supplemented with 0.01% (v/v) acetone (mock) or 1 μM *rac*-GR24, and from 5-day-old *Arabidopsis* seedlings grown under continuous white light on half-strength Murashige and Skoog medium. The harvested material was snap-frozen in liquid nitrogen, and cells were disrupted by 3-mm metal beads in 2-ml tubes (Eppendorf) with a mixer mill 400 (Retsch) for 2 min at 20 Hz. RNA was extracted with the RNeasy Plant Mini Kit (Qiagen), and genomic DNA was removed by DNase treatment. Subsequently, RNA was purified by ammonium acetate (2.5 M final concentration) precipitation. RNA concentrations were measured with a ND-1000 Spectrophotometer (Thermo Fisher Scientific Nanodrop), and 1 μg was reverse transcribed with the iScript cDNA Synthesis Kit (Bio-Rad). The primers were designed with the primer design tool of Roche Applied Science (http://www.roche-applied-science.com). All primers (see supplemental Table S1) were diluted with water to a concentration of 2.5 μM. All qRT-PCR experiments were performed in three technical repeats on 384-multiwell plates and with SYBR Green detection. Reaction mixes were composed by the Janus Robot (PerkinElmer) with a final volume of 5 μl and a 10% cDNA fraction with the SYBR Green Master Mix (PerkinElmer). The Roche Lightcycler 480 system (Roche Diagnostics) was used to execute all qRT-PCR reactions with the following settings: 1× preincubation (95 °C for 5 min), 45× amplification (95 °C for 10 s, 60 °C for 10 s, and 72 °C for 10 s), 1× melting curve (95 °C for 5 s, 65 °C to 97 °C for 1 min), and 1× cooling down (40 °C for 10 s). Threshold cycle and efficiency values were determined by the Lightcycler 480 software and analyzed by the 2-ΔΔCt method (59). The obtained expression data were normalized to the expression levels of *ACTIN2* (*ACT2*, AT3G18780).

### Experimental Design and Statistical Rationale

For each TAP experiment in *Arabidopsis* cell cultures, two independent biological replicates were carried out after treatment with the synthetic SL analog, *rac*-GR24 or 0.01% (v/v) acetone (mock), to determine the reproducibility of the prey identifications from purifications. *Bona fide* interactors were identified by subtracting background proteins from the list of all copurified proteins based on the frequency of occurrence in a large GS TAP data set (42). Three independent biological replications were done for the GFP-trapping experiments in *Arabidopsis* cell cultures expressing KAI2 and D14 as baits. A two-sided Student’s *t* test was used for quantitative comparison with LFQ to detect changes in the protein complex composition between the baits. Three biological repeats were carried out for single-step AP from *35S::GS-MAX2* (Col-0) and *35S::GS-MAX2* (*papp5-1*) seedlings treated with 0.01% (v/v) acetone (mock) or 1 μM *rac*-GR24, followed by phosphopeptide enrichment using TiO_2_ magnetic beads. All physiological assays were done in three biological repeats, and *n* represents the statistical unit as explained below. In the shoot branching assay, *n* = 45 corresponds to three repeats with 15 plants per genotype for which the rosette branches were counted in each repeat; in the hypocotyl elongation assay, *n* = 6 represents two plates per repeat for each genotype and condition with at least 35 seedlings measured for one plate; in the lateral root density assay, *n* = 9 corresponds to three plates per repeat for each condition and genotype with 30 seedlings measured per plate: and in the seed germination assay, *n* = 9 represents three 96-well plates per repeat with 12 wells containing 15 to 40 seeds each per genotype and condition. For the statistical analysis, the Student’s *t* test (shoot branching assay), Poisson regression model (lateral root density assay), ANOVA mixed model with *post hoc* Tukey–Kramer (hypocotyl elongation and seed germination assays), and ANOVA mixed model (qPCR analysis) were used. Bimolecular fluorescence complementation (BiFC), Y2H, and coimmunoprecipitation (Co-IP) experiments were carried out at least in three independent repeats to ensure reproducibility of the results. The synthetic SL analog GR24 is a racemic mixture (*rac*-GR24) comprising equal amounts of (+)-GR24^5DS^ and (−)-GR24^ent-5DS^ enantiomers, which are preferentially perceived by D14 and KAI2, respectively (60). *rac*-GR24 was dissolved to a 10-mM concentration in acetonitrile (seed germination assay) or acetone (other experiments). KAR2 was dissolved to a 10-mM concentration in 70% (v/v) methanol.

## Results

### The MAX2 Pathway is Active in Arabidopsis Cell Cultures and GS-Tagged Baits are Functional in Planta

*Arabidopsis* cell suspension cultures were chosen for TAP experiments owing to the high protein yield and the possibility to carry out trigger-based studies (42). To this end, translational fusions were made between the protein of interest (bait) and a double-affinity GS tag, consisting of two IgG-binding domains of the G protein and a streptavidin-binding peptide, separated by a tobacco etch virus protease cleavage site (61). This system has already been used to characterize the signaling complexes of many hormonal pathways, including auxin (62), abscisic acid (63), jasmonate (64, 65), and recently SLs (44). Previously, the response of cell cultures expressing *35S::GSrhino-SMXL7* toward the SL analog *rac*-GR24 has been tested on the protein level, with degradation of the bait as a result (44). To examine the response toward the SL analog in our system, the wild-type cell cultures were treated with *rac*-GR24 and the expression level of one of the SL-responsive genes, *BRC1*, was measured at various time points by qRT-PCR. The increase in the relative expression level of *BRC1* was detected over the course of the *rac*-GR24 treatment, reaching a 10-fold increase at 5 h (supplemental Fig. S1), demonstrating that the downstream MAX2 signaling is active.

**Fig. 1:**
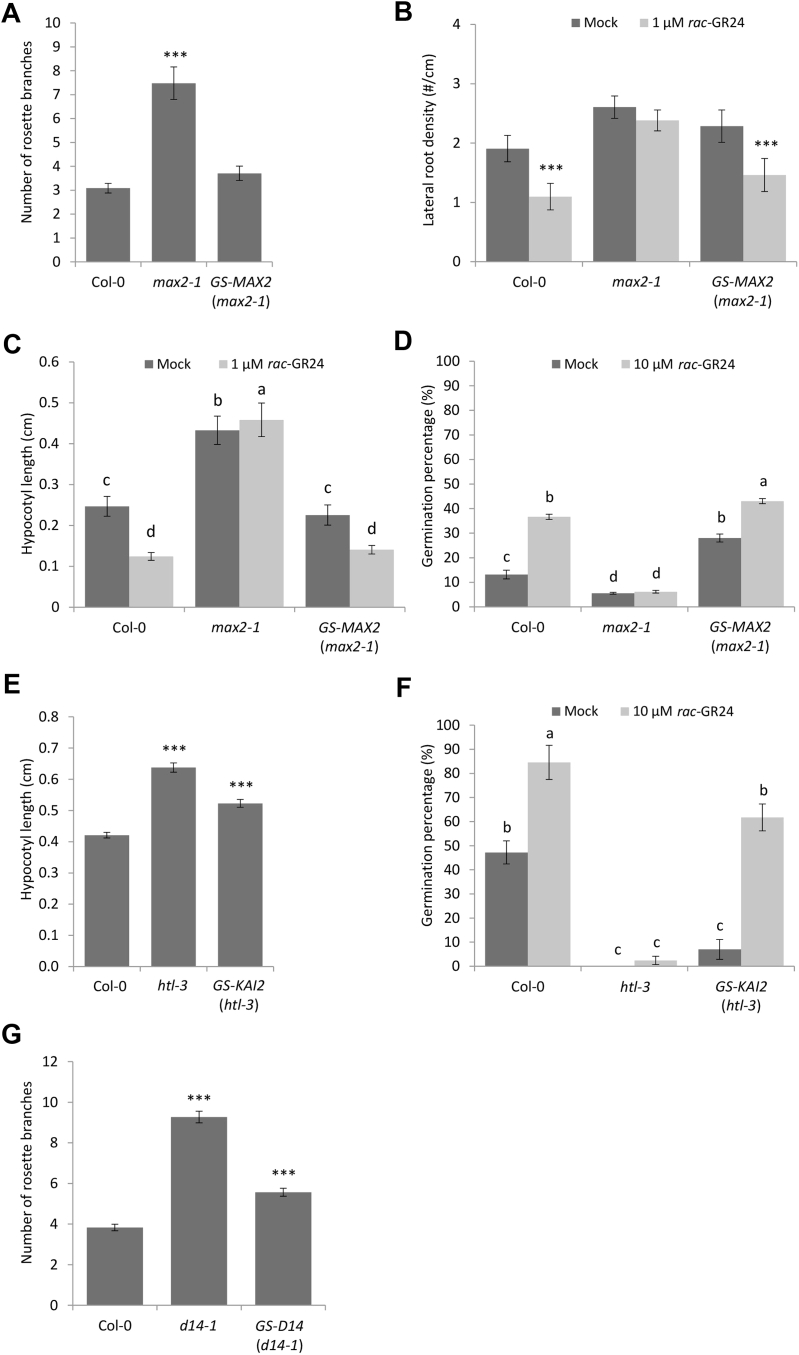
**Phenotypes of the corresponding mutants complemented by the GS-tagged MAX2, D14, and KAI2.** Rosette branches of Col-0, *max2-1*, and *35S::GS-MAX2* (*max2-1*) (*A*) or of Col-0, *d14-1*, and *35S::GS-D14* (*d14-1*) (*G*) lines counted after 6 weeks of growth (*n* = 45, representing three repeats with 15 plants per genotype per repeat). *B*, lateral root density of Col-0, *max2-1*, and *35S::GS-MAX2* (*max2-1*) lines analyzed in 9-day-old plants (*n* = 9, representing three repeats with three plates per repeat and 30 seedlings per plate). Hypocotyl length measured in Col-0, *max2-1*, and *35S::GS-MAX2* (*max2-1*) (*C*) or Col-0, *htl-3*, and *35S::GS-KAI2* (*htl-3*) (*E*) lines grown in continuous red light for 4 days (*n* = 6, representing three repeats with two plates per repeat and at least 35 seedlings per plate). Seed germination assay of Col-0, *max2-1*, and *35S::GS-MAX2* (*max2-1*) (*D*) or Col-0, *htl-3*, and *35S::GS-KAI2* (*htl-3*) transgenic lines (*E*). Seeds were distributed in 96-well plates containing Hepes buffer with mock (acetonitrile) or 10 μM *rac*-GR24 and placed for 5 days at 24 °C in the dark (*n* = 9, corresponding to three repeats with three 96-well plates per repeat with 12 wells containing 15–40 seeds each). *Asterisks* indicate statistically significant differences between genotypes and Col-0 (*A*, *E*, and *G*) or treatments (*B*) (∗∗∗*p* < 0.001, Student’s *t* test [*A*, *E*, and *G*], Poisson regression model [*B*]). *C*, *D*, and *F*, statistical groupings (letters a-d) were determined by ANOVA mixed model with Tukey–Kramer honestly significant difference (*p* < 0.05). Values are means with error bars representing the standard error (SE).

In addition, we tested whether the GS tag did not interfere with the function of the bait proteins. To this end, transgenic *Arabidopsis* plants were generated expressing *35S::GS-MAX2*, *35S::GS-D14*, and *35S::GS-KAI2* in the corresponding mutant backgrounds. For each construct, one line with high expression of the tagged protein was selected for further physiological studies (supplemental Fig. S2*A*). The GS-tagged MAX2 protein rescued several previously described phenotypes of the *max2-1* mutant, including extensive shoot branching, elongated hypocotyl of red-light-grown seedlings, and low seed germination frequency under suboptimal temperature conditions (Fig. 1, *A*, *C* and *D*). Furthermore, the sensitivity to the *rac*-GR24 treatment of *max2-1* mutants in the lateral root density, hypocotyl elongation, and seed germination assays was restored in the *35S::GS-MAX2* (*max2-1*) transgenic lines (Fig. 1, *B*–*D*). For the D14 and KAI2 complementation, only the phenotypes that are specific for each of the pathways were tested. The GS-tagged D14 partially complemented the high number of shoot branches of the *d14-1* mutant (Fig. 1*G*). Similarly, introduction of the *35S::GS-KAI2* construct partially reduced the elongated hypocotyl of red-light-grown *htl-3* plants (Fig. 1*E*) and restored the mutant sensitivity toward *rac*-GR24 in the seed germination assay (Fig. 1*F*). These findings indicate that the GS-tagged MAX2 fully complements the tested phenotypes of the *max2-1* mutant, whereas for the GS fusions with the D14 and KAI2 proteins only a partial complementation was observed of the phenotypes of the respective mutants.

### Affinity Purification Resolves Components of MAX2, D14, and KAI2 Complexes

To unravel the MAX2, KAI2, and D14 protein complexes, *Arabidopsis* cell suspension cultures were stably transformed with the N-terminally GS-tagged bait under control of the 35S promoter, by *A. tumefaciens*-mediated cocultivation (61). In addition, we created a truncated MAX2 protein version, *i.e.*, MAX2ΔFBOX, of which the first 53 amino acids were deleted to prevent the interaction with SKP1, and therefore the entire SCF E3 ligase complex (1). This modified F-box protein is predicted to create a stable complex with the substrate that can be bound but is not targeted for ubiquitination (5, 66). By using the full-length protein and truncated version we expected to detect ubiquitination-dependent interactors of MAX2. The expression of the baits in the cell cultures was checked by immunoblotting, and high protein levels were detected for all constructs (supplemental Fig. S2*B*). The cell material was harvested 5 h after treatment with either 1 μM *rac*-GR24 or 0.01% (v/v) acetone (mock). After the protein extraction, a two-step purification on IgG and streptavidin beads was done as described (42).

To identify *bona fide* interactors, the retrieved MS/MS spectra were analyzed according to a well-established, qualitative procedure, in which proteins that were present in a list of nonspecific and sticky binders (42) were filtered out from the dataset. As a result, 19 proteins were identified as potential preys of MAX2 (Table 1, supplemental Dataset 1), and STRING analysis, based on the experimental evidence, revealed a complex interaction network between them (supplemental Fig. S3). As the F-box protein MAX2 is known to be part of an E3 ligase, general SCF complex proteins (AT5G42190/ASK2, AT4G02570/CUL1, AT1G20140/ASK4, AT1G75950/SKP1, and AT3G60010/ASK13) were identified. Moreover, six of the nine members of the CONSTITUTIVE PHOTOMORPHOGENIC 9 (COP9) signalosome (CSN) (67) were also copurified with MAX2 (AT3G61140/CSN1, AT2G26990/FUS12, AT5G14250/COP13, AT5G42970/COP8, AT1G22920/CSN5A, and AT5G56280/CSN6A). As expected, the SCF components were not copurified with MAX2ΔFBOX, indicating that the deletion present in that construct abolished the interaction with the E3 ligase complex. Besides, a subunit of nuclear DNA-dependent RNA polymerases II (AT2G15430/RPB35.5A), serine/threonine protein phosphatase (AT2G42810.1/PAPP5), ubiquitin-like superfamily protein (AT5G25270.1/ubiquitin-like protein), proteins involved in folding (AT3G22480/PFD2, AT5G49510/PFD3, and AT3G25230/ROF1), and plant innate immunity (AT4G11260/ATSGT1B) were detected as interactors. One protein with an unknown function was isolated as well (AT5G54550). No peptides could be detected for the SMXL proteins, which are known targets of the MAX2-D14 complex for ubiquitination and 26S-proteasomal degradation (18, 33, 35). In our experimental setup, the association between MAX2 and D14 or KAI2 receptors could not be recovered as well (56, 68, 69).

**Table 1:** Overview of prey proteins identified by TAP with MAX2 and MAX2ΔFBOX as bait

AGI	Protein	MAX2		MAX2ΔFBOX	
		Mock	*rac*-GR24	Mock	*rac*-GR24
AT2G42620.1	MAX2	2	2	2	2
AT5G42190.1	ASK2	2	2	0	0
AT1G20140.1	ASK4	2	2	0	0
AT5G14250.1	COP13	2	2	0	0
AT5G42970.1	COP8	2	2	0	0
AT3G61140.1	CSN1	2	2	0	0
AT1G22920.1	CSN5A	2	2	0	0
AT5G56280.1	CSN6A	2	2	0	0
AT4G02570.1	CUL1	2	2	0	0
AT2G26990.1	FUS12	2	2	0	0
AT1G75950.1	SKP1	2	2	0	0
AT5G54550.1	unknown	2	2	0	0
AT3G60010.1	ASK13	2	1	0	0
AT2G42810.1	PAPP5	2	0	2	2
AT3G22480.1	PFD2	0	0	2	2
AT5G49510.1	PFD3	0	0	2	2
AT4G11260.1	ATSGT1B	0	0	1	2
AT2G15430.1	RPB35.5A	0	0	1	2
AT5G25270.1	Ubiquitin-like protein	0	0	1	2
AT3G25230.1	ROF1	0	0	0	2

AGI, Arabidopsis Genome Identifier.

TAP experiments were done with cell cultures expressing *35S::GS-MAX2* or *35S::GS-MAX2ΔFBOX* treated with 0.01% (v/v) acetone (mock) or 1 μM *rac*-GR24. Prey proteins were identified with peptide-based homology analysis of mass spectrometry data. Background proteins were withdrawn based on the occurrence frequency of copurified proteins in a large GS TAP data set (40). Only prey proteins identified at least twice in all experiments are presented with the number of times they occurred in two experiments with the bait protein for each condition. A detailed list can be found in supplemental Dataset 1.

**Fig. 2:**
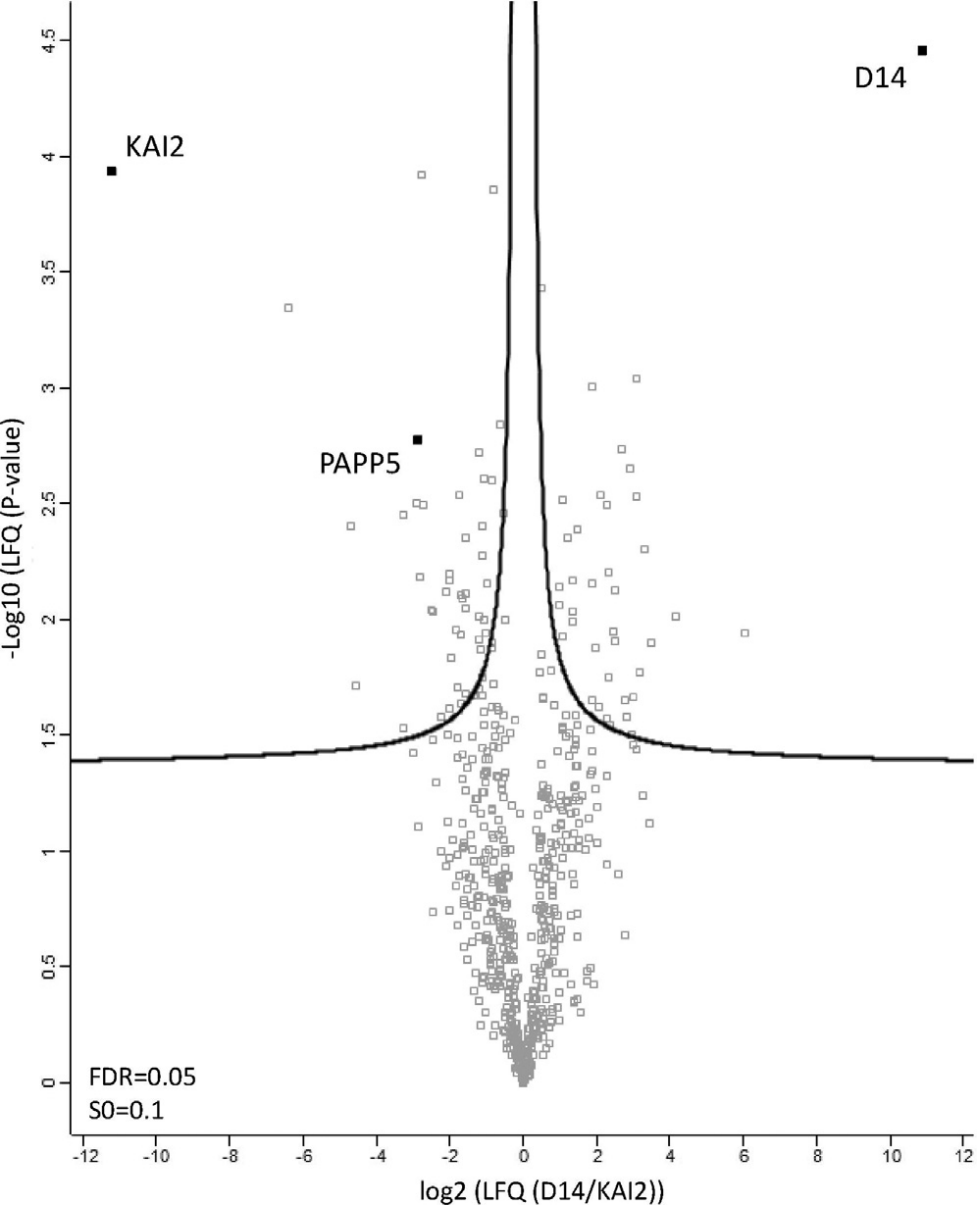
**Differential protein complexes formed around D14 and KAI2 in *Arabidopsis* cell cultures**. Volcano plot showing the distribution of all quantified proteins after filtering and statistical analysis based on LFQ values, with their corresponding protein abundance ratios (KAI2/D14) over the Student’s *t* test *p* value (FDR = 0.05, S0 = 0.1). The cutoff curve indicates which proteins are significantly more associated with D14 (*right*) and KAI2 (*left*).

No putative preys were identified after filtering the background proteins in the TAP experiments with both KAI2 and D14 baits. As weak, low-abundant, or transient protein–protein interactions might be missed owing to the two-step procedure, we used a single-step AP of D14 and KAI2 in combination with an LFQ analysis. By quantitative comparison of the D14 and KAI2 interactomes, we aimed to identify proteins that play a specific role only in one of the signaling pathways. To this end, *Arabidopsis* cell cultures expressing *35S::D14-GFP* and *35S::KAI2-GFP* (supplemental Fig. S2*C*) were treated for 15 min with 1 μM *rac*-GR24. Proteins were isolated in a single purification step using GFP-Trap and identified with LC-MS/MS. Next, spectra were searched with the MaxQuant software and MaxLFQ was used to quantify proteins identified with D14 and KAI2 over the three replicates (70). LFQ values were normally distributed and the Pearson correlation coefficient between all samples ranged from 0.719 to 0.935 (supplemental Fig. S4), demonstrating a good reproducibility between them. Subsequent statistical analysis allowed the identification of prey proteins that were enriched with one of the tested baits. Samples were divided into either “D14” or “KAI2” groups, with each group containing three biological repeats, followed by a Student’s *t* test on the LFQ intensity values. Although we cannot exclude that the list of candidate interactors might contain false positives, 25 and 20 proteins significantly more associated with KAI2 and D14, respectively, were retrieved (Fig. 2; supplemental Table S2; supplemental Dataset 2). Of interest, two proteins significantly more associated with KAI2, AT2G42810.1/PAPP5 and AT3G25230/ROF1, were also identified as putative preys of MAX2 by means of TAP. In contrast, no overlap was found between proteins copurified with MAX2 and those that were significantly more associated with D14. Nevertheless, the interaction between D14 and PAPP5 cannot be disregarded. In two of three AP-MS experiments with D14 as a bait, PAPP5 peptides were detected but at an intensity lower than that in the KAI2 samples (supplemental Dataset 2). Among all proteins identified with KAI2 and D14 we did not detect the previously described interactors, MAX2 and SMXL proteins (18, 35, 56, 68).

### Identification of the PAPP5 Protein

We further focused on PHYTOCHROME-ASSOCIATED PROTEIN PHOSPHATASE5 (PAPP5) because it was detected as a putative prey in both TAP of MAX2 and AP experiments with KAI2 and D14. The *PROTEIN PHOSPHATASE 5* (*PP5*) gene in *Arabidopsis* and *Solanum lycopersicum* (tomato) was shown to be alternatively spliced, resulting in two protein isoforms that are differently localized in the cell. One of both transcripts was translated into a large PP5 isoform owing to the presence of an additional exon that encodes two putative transmembrane domains. Studies in tomato demonstrated that this larger isoform was an integral membrane protein that was targeted to the endoplasmic reticulum and nuclear envelope, whereas the smaller isoform was soluble and localized in the nucleus and cytoplasm (71). The latter isoform is referred to as PAPP5, because of its interaction with phytochromes, thereby increasing their stability and activity (57). The MS analysis provided evidence that the latter splice variant is present in our dataset, because a unique peptide of AT2G4281.1 could be identified (supplemental Fig. S5, supplemental Dataset 1).

**Fig. 3:**
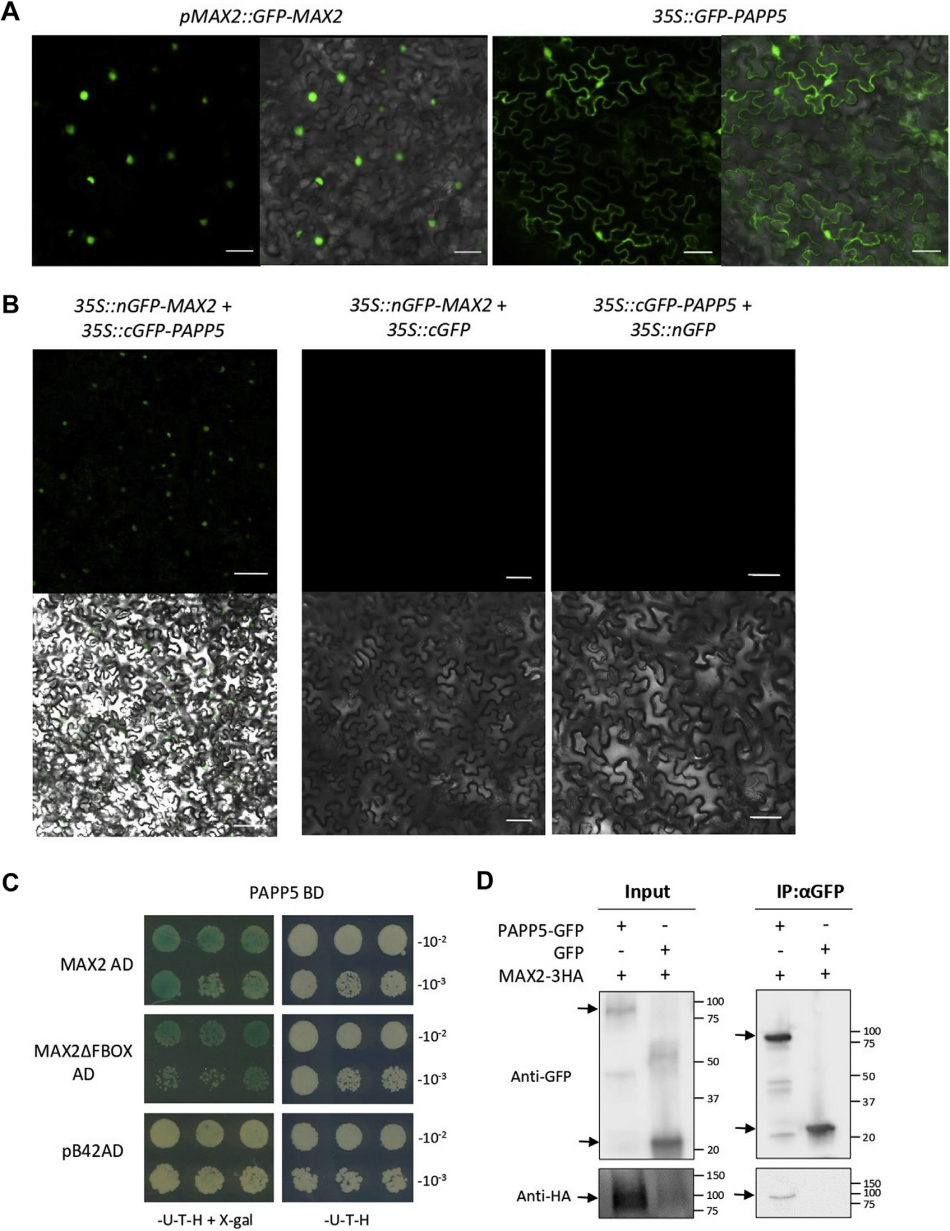
**PAPP5-MAX2/MAX2ΔFBOX protein localization and interactions.***A*, subcellular localization of MAX2 and PAPP5. *N. benthamiana* leaves were transiently transformed with *pMAX2::GFP:MAX2* (*left*) and *35S::GFP:PAPP5* (*right*). The scale bar represents 20 μm. *B*, BiFC analysis of the MAX2 and PAPP5 interaction. *N. benthamiana* leaves were transiently transformed with *35S::nGFP-MAX2* and *35S::cGFP-PAPP5* fusion constructs. Each construct was coexpressed with the corresponding GFP part (*35S::cGFP* or *35S::nGFP*) as a negative control. GFP signal only (*top*) or merged with bright-field images (*bottom*) are shown. The scale bar represents 20 μm. *C*, Y2H screen of the MAX2 and PAPP5 interaction. PAPP5 fused to GAL4-BD was tested for the interaction with MAX2 and MAX2ΔFBOX fused to GAL4-AD or pB42AD for negative control. Yeast transformed with both plasmids was selected on SD (Raf/Gal)-U-T-H medium supplemented with X-Gal, and the interaction was scored based on the *blue coloring* of the colonies. *D*, *in vivo* interaction between PAPP5-GFP and MAX2-3HA revealed by the Co-IP assay. Protein extracts were prepared from *N. benthamiana* leaves transiently expressing *35S::PAPP5-GFP* and *35S::MAX2-3HA*. The *35::GFP* construct was cotransformed with *35S::MAX2-3HA* as a negative control. Input means total protein lysate without immunoprecipitation. Molecular masses: 87.2 kDa, PAP5-GFP; 80.4 kDa, MAX2-3HA; 26.9 kDa, GFP. All experiments were repeated three times with comparable results.

PAPP5 was copurified with MAX2ΔFBOX under both conditions while the interaction with full-length MAX2 was not found in the presence of *rac*-GR24. As PAPP5 and MAX2 need to be present in the same cellular compartment to interact, we investigated the localization patterns of both proteins. Translational fusions of MAX2 and PAPP5 (splice variant AT2G42810.1) with GFP were transiently expressed in *N. benthamiana* leaves. The N-terminal GFP fusion of MAX2, under control of its own promoter, localized in the nucleus, which was in agreement with previous observations (36, 72) (Fig. 3*A*). As the N-terminal GFP fusion of PAPP5 under control of its own promoter did not result in a clear signal, we analyzed this fusion protein under the control of the constitutive *35S* promoter. PAPP5 localized in both the nucleus and the cytoplasm, similarly as described (57, 73, 74) (Fig. 3*A*). In conclusion, MAX2 and PAPP5 have an overlapping localization pattern in the nucleus. To confirm the AP results, we tested the MAX2-PAPP5 interaction by means of various methods. First, we used a LexA-based Y2H assay to examine whether the interaction between the proteins was direct. In our screen, yeast cotransformed with PAPP5 and MAX2 or MAX2ΔFBOX fused to the GAL4-BD or GAL4-AD, respectively, stained blue on the selective medium SD (Raf/GAL)-Ura-Trp-His (-U-T-H) supplemented with X-Gal, indicating that the tested proteins interacted (Fig. 3*C*). Then, we set up a BiFC assay in *N. benthamiana* leaves to detect the MAX2-PAPP5 interaction site in the cell. To this end, fusion proteins were generated with either the N-terminal (nGFP) or C-terminal (cGFP) fragment of GFP. Upon the interaction between the tested proteins, these GFP fragments could reassemble, with fluorescence as a consequence. To further confirm the MAX2 and PAPP5 association, different combinations of N- and C-terminal fusions with nGFP and cGFP were tested by transient coexpression in *N. benthamiana* leaves. MAX2 and PAPP5 interacted in the nucleus when the N-terminal nGFP fusion protein of MAX2 was combined with the N-terminal cGFP fusion protein of PAPP5 (Fig. 3*B*). In addition, Co-IP experiments in *N. benthamiana* leaves demonstrated that MAX2-3HA copurifies with PAPP5-GFP (Fig. 3*D*). Together, these results confirmed the direct interaction between MAX2 and PAPP5 in the nucleus.

**Fig. 4:**
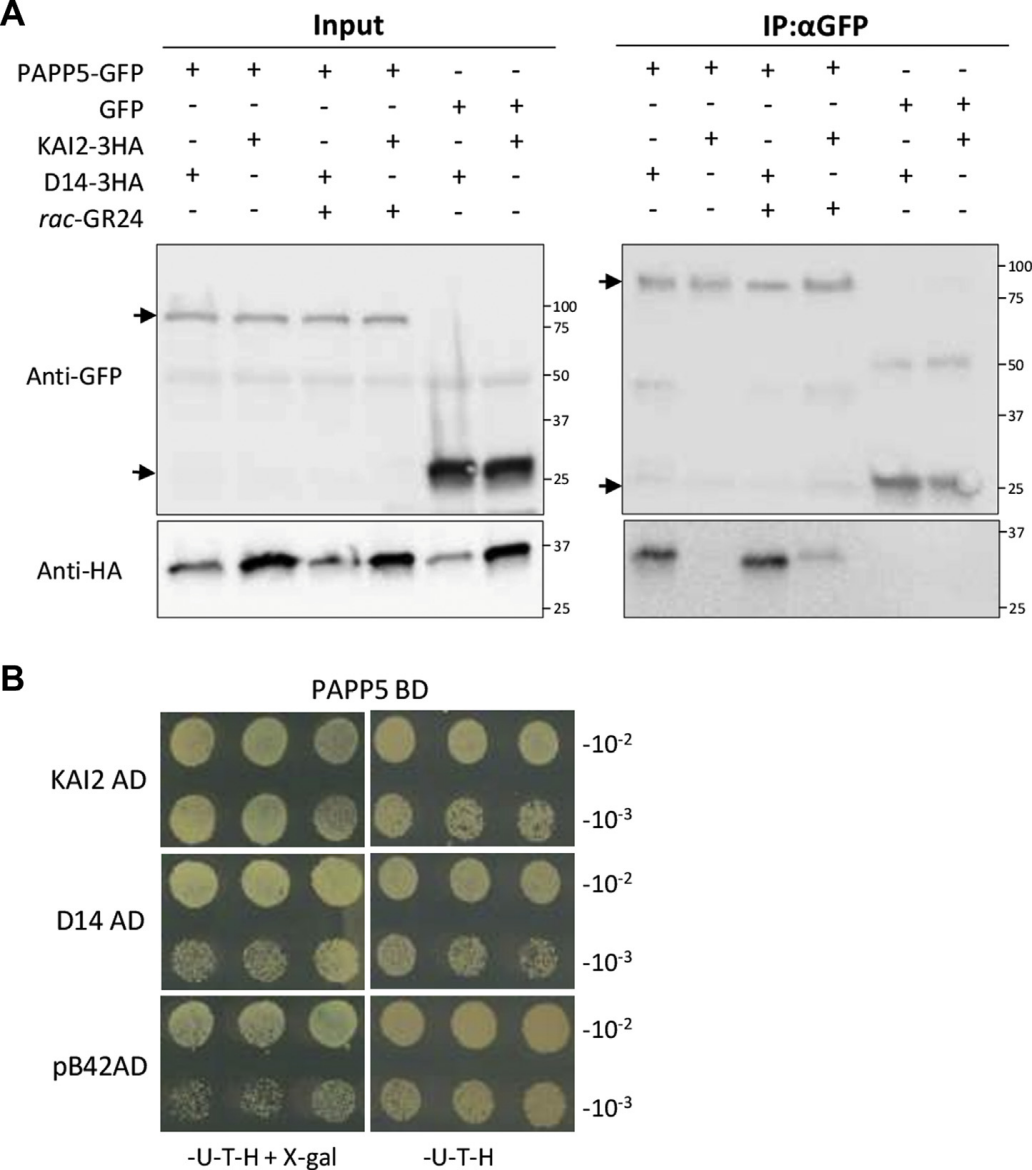
**PAPP5 belonging to both KAI2 and D14 signaling complexes.***A*, *in vivo* interaction between PAPP5-GFP and KAI2-3HA or D14-3HA revealed by the co-IP assay. Protein extracts were prepared from *N. benthamiana* leaves transiently expressing *35S::PAPP5-GFP* and *35S::KAI2-3HA* or *35S::D14-3HA* under mock conditions or treated with 10 μM *rac*-GR24 for 30 min. The *35S::GFP* construct was cotransformed with *35S::KAI2-3HA* or *35S::D14-3HA* as a negative control. Input means total protein lysate without immunoprecipitation. Molecular masses: 87.2 kDa, PAP5-GFP; 32.6 kDa, D14-3HA; 32.8 kDa, KAI2-3HA; 26.9 kDa, GFP. *B*, Y2H screen of the PAPP5 and KAI2/D14 interaction. PAPP5 fused to GAL4-BD was tested for interaction with KAI2 and D14 fused to GAL4-AD or pB42AD for negative control. Yeast transformed with both plasmids was selected on SD (Raf/Gal)-U-T-H medium supplemented with X-Gal, and the interaction was scored based on the *blue coloring* of the colonies. All experiments were repeated three times with comparable results.

Next, we tried to address the question whether PAPP5 interacts with MAX2 both during KAI2 and D14 signaling or is specific for one of the pathways. In *rac*-GR24-treated *Arabidopsis* cell cultures, PAPP5 was more involved in KAI2 protein complexes than with those of D14, although it was also copurified with D14 in two of three AP-MS repeats. For that reason, we further investigated the interaction between PAPP5, KAI2, and D14 by means of Co-IP in *N. benthamiana* leaves. It is remarkable that KAI2-3HA interacted with PAPP5-GFP only after 15 min of treatment with *rac*-GR24, whereas D14-3HA copurified with PAPP5-GFP under both conditions (Fig. 4*A*). To test whether PAPP5 interacts directly with KAI2 and D14, we carried out a Y2H LexA assay in which PAPP5 fused to GAL4-BD was tested with both KAI2 and D14 fused to GAL4-AD. Neither KAI2-AD nor D14-AD cotransformed with PAPP5-BD in yeast cells could activate the expression of the reporter gene to allow blue staining, without influence of *rac*-GR24 on the results (Fig. 4*B*). Taken together, PAPP5 seems to be an indirect interactor of both receptors.

### PAPP5 Protein and Transcript Levels are not Influenced by *rac*-GR24

The F-box protein MAX2 is expected to regulate ubiquitination and degradation of specific targets in response to *rac*-GR24, such as the SMXL proteins (32, 34, 35). To test whether the stability of the PAPP5 protein might also be affected by the SL analog, cell suspension cultures expressing *35S::GS-PAPP5* were treated with 0.01% (v/v) acetone (mock) or with 1 μM *rac*-GR24. Samples were harvested before and at six different time points after treatment (1 min and 1, 5, 10, 25, and 50 h). No differences in protein abundance could be observed at any time point (supplemental Fig. S6*A*) by means of Western blot, suggesting that PAPP5 is not degraded in response to *rac*-GR24. We then used qRT-PCR to investigate changes in *PAPP5* expression levels in wild-type cell cultures treated with 0.01% (v/v) acetone (mock) or with 1 μM *rac*-GR24 for 1, 5, 10, or 15 h, but no differences could be detected as well (supplemental Fig. S6*B*). Taken together, *rac*-GR24 treatment does not affect PAPP5 on the protein or the transcript level in *Arabidopsis* cell cultures.

### MAX2 Might Be a Target for PAPP5 Phosphatase Activity

**Fig. 5:**
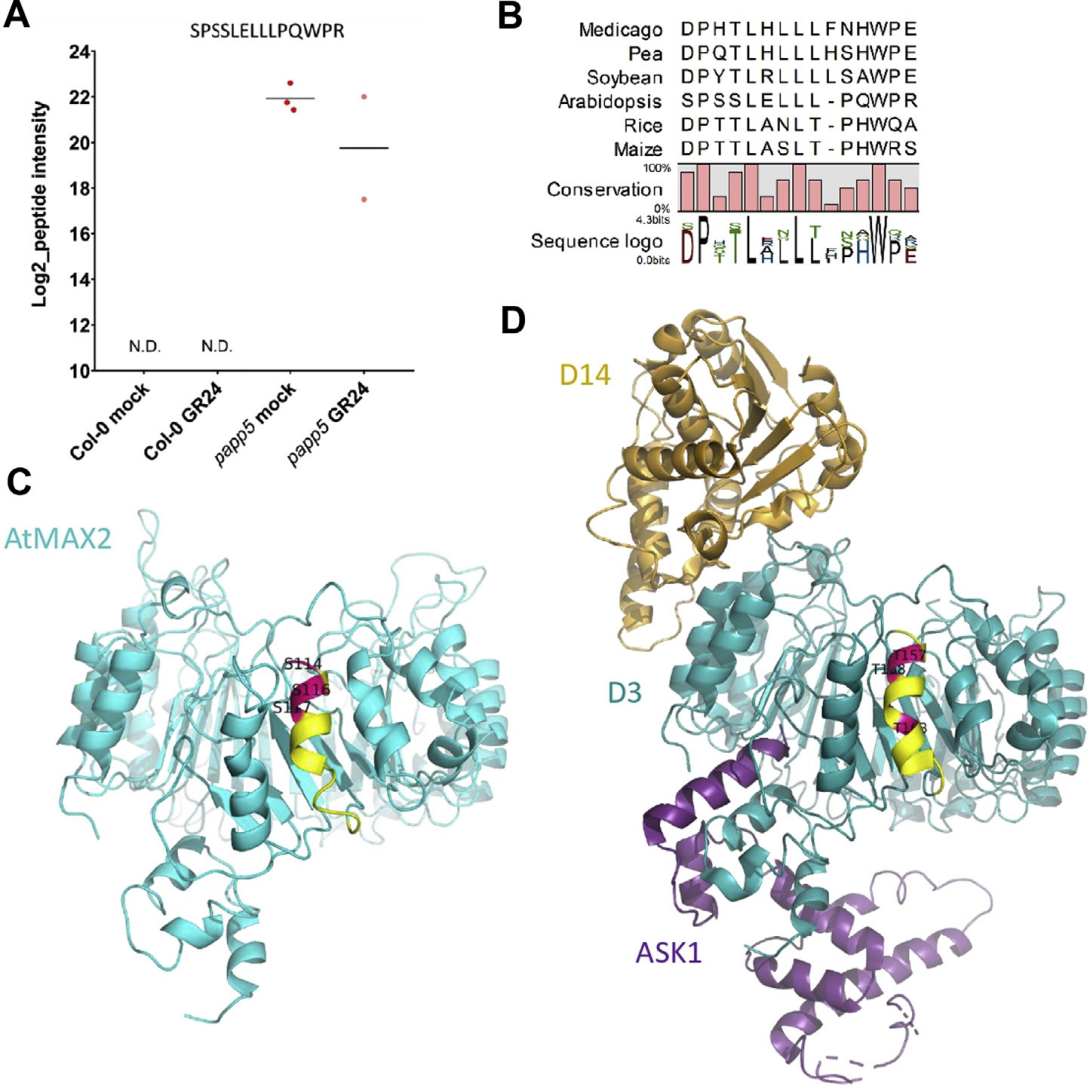
**Identified phosphopeptide of MAX2.***A*, Log2-Intensity of the monophosphorylated phosphopeptide SPSSLELLLPQWPR of MAX2. Each dot represents one biological replicate. The average log2-Intensity is represented by *horizontal black line*. N.D., not detected. Identified phosphopeptide of MAX2 is solvent exposed. *B*, alignment of MAX2 phosphopeptide identified in *Arabidopsis* (NP_565979) with the MAX2 orthologs from rice (*O. sativa*, accession XP_015643693), medicago (*Medicago truncatula*, XP_003607592), pea (*Pisum sativum*, ABD67495), soybean (*Glycine max*, XP_003540983), and maize (*Zea mays*, XP_020394883). The graphic view of alignment was generated by CLC Main workbench 8.1.2 using multiple sequence alignment of protein sequences. *C*, the protein model of AtMAX2, generated with the SWISS-MODEL webserver using the D3 crystal structure (PDB: 5HZG) and the AtMAX2 protein sequence. *D*, the crystal structure of the SL-induced AtD14-D3-ASK1 complex (PDB: 5HZG). AtD14, D3, and ASK1 are colored in *orange*, *blue*, and *purple*, respectively. The identified phosphorylated peptide is indicated in *yellow*, and the potentially phosphorylated serines/threonines are in *pink*.

Posttranslational modifications, such as phosphorylation, can modulate protein function, localization, or activity (75). Thus far no posttranslational modifications of *Arabidopsis* MAX2 have been identified. PAPP5 is a type 5 serine/threonine protein phosphatase and has previously been described to interact and dephosphorylate the biologically active far-red light–absorbing phytochrome A (57). To understand the relationship between MAX2 and PAPP5, we evaluated whether PAPP5 is capable of dephosphorylating MAX2. Therefore, we carried out the single-step AP of MAX2 overexpressed in Col-0 or *papp5-1* mutant backgrounds (supplemental Fig. S2*D*) treated with 0.01% (v/v) acetone (mock) or 1 μM *rac*-GR24 for 15 min. For each condition, three biological repeats were sampled. Proteins were extracted and digested with trypsin/Lys-C after the single-step purification with magnetic IgG beads, and phosphopeptides were enriched with TiO_2_ magnetic beads. After LS-MS/MS analysis, a database search revealed a MAX2 phosphopeptide (S^114^PS^116^S^117^LELLLPQWPR) in the *papp5-1* but not in the Col-0 background (Fig. 5*A*, supplemental Dataset 3). Although the phosphopeptide of MAX2 was identified with high confidence (PEP < 0.001), the phosphosite S116 was detected with high localization probability (0.91) in only one of the *papp5-1* samples, but no exact phosphosite could be identified in the other repeats. The structure of AtMAX2 modeled based on the rice D3 crystal structure as a template (26) revealed that two serine residues S114 and S116 are exposed to the solvent and, therefore, possibly phosphorylated (Fig. 5*C*). The phosphorylated peptide identified for *Arabidopsis* seemed to be conserved in MAX2 orthologs in the selected species (for the percentage of conservation, see Fig. 5*B*) with several potential phosphosites (Ser or Thr). The most conserved residue S117, however, is not exposed to the solvent in our AtMAX2 model. Furthermore, the identified phosphopeptide seems not to be blocked by the interaction with D14 and ASK1, when modeled on the crystal structure of the SL-induced AtD14-D3-ASK1 complex (26) (Fig. 5*D*). No difference in MAX2 phosphorylation was observed due to the *rac*-GR24 treatment neither in the Col-0 nor in the *papp5-1* mutant background (Fig. 5*A*). Hence, our results suggest that MAX2 is a substrate for the PAPP5 phosphatase activity independently of *rac*-GR24.

### *PAPP5* is not Involved in Shoot Branching Control and Lateral Root Development

To investigate the role of *PAPP5*, we assessed different phenotypes and responses toward *rac*-GR24 in the *papp5* mutants. Two independent transfer DNA (T-DNA) insertion alleles in the *PAPP5* gene in the Col-0 accession were isolated. The *papp5-1* loss-of-function mutant carrying the mutation in the 5’ untranslated region (UTR) has been described (57) (supplemental Fig. S7*A*). The *papp5-3* mutant insertion lies in the seventh intron (supplemental Fig. S7*A*) ahead of the region encoding the predicted phosphatase domain. The *PAPP5* transcript levels assessed by qRT-PCR increased ahead of the T-DNA insertion and significantly reduced behind the insertion, suggesting that the function of the phosphatase domain might be disturbed (supplemental Fig. S7*B*). In addition, double mutants of *papp5-1* and the receptors *d14-1* or *htl-3* were generated to evaluate specific SL or KAR/KL-related outcomes, respectively.

Originally, SLs were discovered as plant hormones because of their impact on shoot branching (6, 7). The potential role of *PAPP5* in this phenotype was examined by counting the number of rosette branches in the *papp5-1* plants compared with that in Col-0, *d14-1*, and the *papp5-1 d14-1* double mutant after 6 weeks of growth. The average number of rosette branches for *papp5-1* did not differ from that of Col-0, indicating that the SL signaling was not disturbed (supplemental Fig. S8*A*). In agreement with previous reports, the number of rosette branches in the *d14-1* mutants was higher than that in the wild-type plants (11, 24, 55) and this phenotype remained unchanged in the *papp5-1 d14-1* double mutant (supplemental Fig. S8*A*).

Besides their role in shoot branching, SLs have been proposed to function in lateral root development (14, 16, 76), but recently, also the KAR/KL pathway has been found to contribute to the control of this phenotype (17). The SL analog *rac*-GR24 decreases the lateral root density in wild-type plants but not in the *max2* mutant (14, 16). To assess whether *PAPP5* is involved in this response, we measured the lateral root density in 9-day-old Col-0, *papp5-1*, *papp5-3, d14-1*, *papp5-1 d14-1*, *htl-3*, and *papp5-1 htl-3* plants grown on half-strength Murashige and Skoog medium supplemented either with 1 μM *rac*-GR24 or 0.01% (v/v) acetone. Under mock conditions, both *papp5* mutants had a phenotype similar to that of Col-0. The sensitivity to the *rac*-GR24 treatment was slightly, but significantly, lower in *papp5-1* plants than that in the wild-type (*p* < 0.05) and comparable with that of the *d14-1* and *htl-3* mutants (supplemental Fig. S8, *B* and *C*), whereas the response of the *papp5-3* mutant did not differ from that of Col-0 (supplemental Fig. S9*A*). Even despite the lack of significant difference in the percentage decrease in the lateral root density between the double mutants *papp5-1 d14-1* and *papp5-1 htl-3* and the single receptor mutants (supplemental Fig. S8, *B* and *C*), the data suggest that *PAPP5* does not play a major role in the SL and KAR/KL pathways to control this response. In conclusion, *PAPP5* is seemingly not involved in shoot branching control and lateral root development.

### *PAPP5* Regulates Seedling Development

**Fig. 6:**
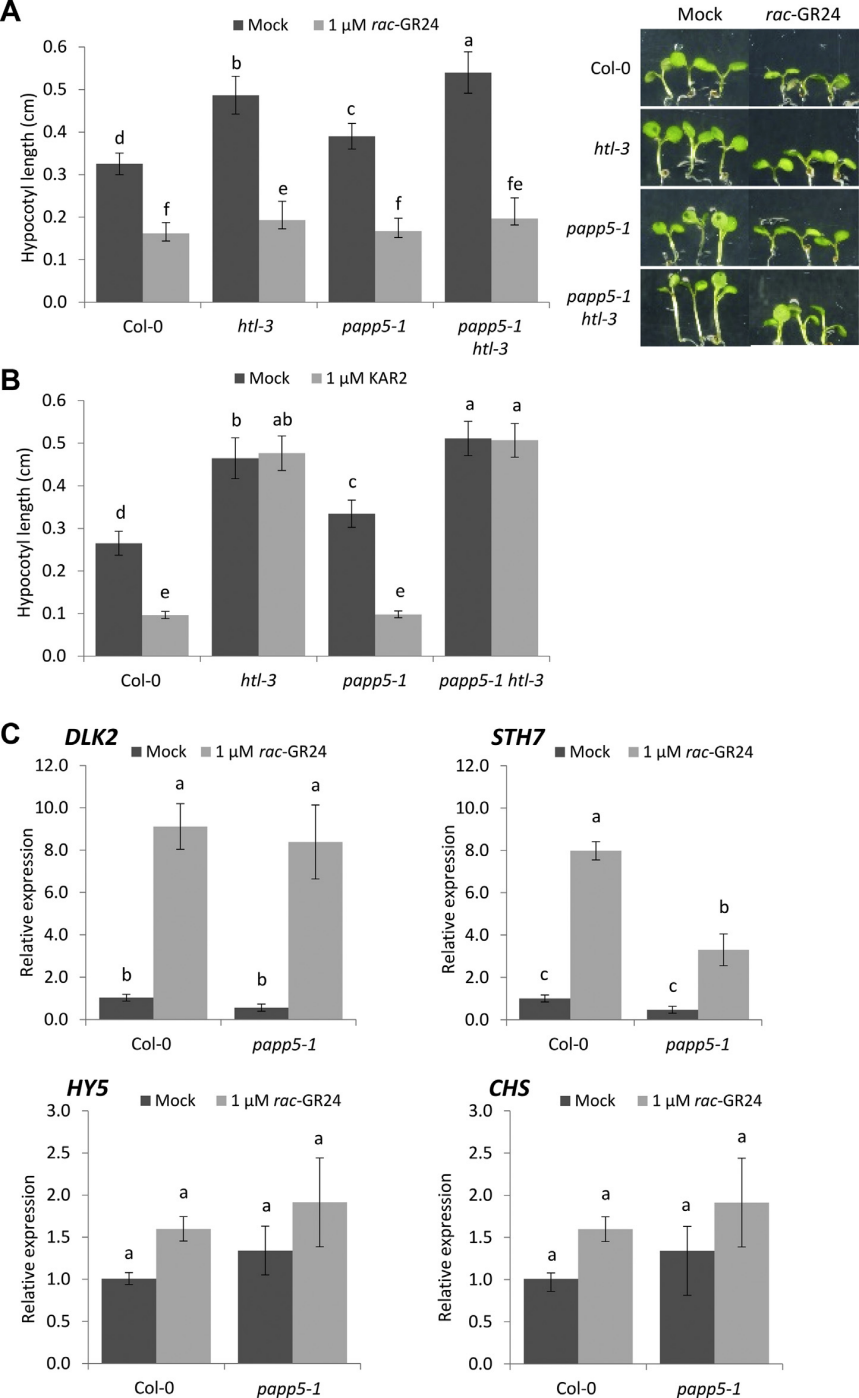
**Young seedling growth and *rac*-GR24 responses modulated by *PAPP5*.***A*, hypocotyl length measured in seedlings grown in red light for 4 days on half-strength Murashige and Skoog medium without sucrose, supplemented with 0.01% (v/v) acetone (mock) or 1 μM *rac*-GR24 (*A*) and 0.01% (v/v) methanol (mock) or 1 μM KAR_2_ (*B*) (*n* = 6, representing three repeats with two plates per repeat and at least 35 seedlings per plate). *C*, expression of KAR/SL transcriptional markers detected by qRT-PCR in Col-0 and *papp5-1* seedlings grown for 4 days in the continuous red light on medium supplemented with 1 μM *rac*-GR24 or 0.01% (v/v) acetone (mock). Expression values are relative to the *ACN2* reference gene. Error bars represent the standard error (SE), based on three independent biological repeats. Statistical groupings (letters a-f) were determined by ANOVA mixed model with Tukey–Kramer honestly significant difference (*p* < 0.01).

In continuous red light, the hypocotyl of *max2-1* and *htl-3* mutants is longer than that of wild-type plants, whereas the hypocotyl length of *d14-1* mutants does not differ from that of Col-0. In addition, *rac*-GR24 is known to decrease the hypocotyl length under these conditions in wild-type and *htl-3* plants but not in the *max2-1* mutant (11). In agreement, in our experiment, the hypocotyl of Col-0 and the mutants *htl-3*, *papp5-1*, *papp5-3*, and *papp5-1 htl-3* was shorter upon *rac*-GR24 treatment than that under mock conditions (Fig. 6*A*, supplemental Fig. S9*B*). Treatment with KAR2, as expected, decreased the hypocotyl length in wild-type and all tested mutants, except in *htl-3* and *papp5 htl-3* (Fig. 6*B*). Of interest, similarly as for *htl-3*, a significantly longer hypocotyl was measured for the *papp5-1* and *papp5-3* mutants under mock conditions, implying that *PAPP5* is necessary for normal seedling development (Fig. 6, *A* and *B*, supplemental Fig. S9*B*). The hypocotyl length of the *papp5-1 htl-3* double mutant was slightly, but significantly, longer than that of each of the single mutants when grown under mock conditions (Fig. 6, *A* and *B*). Furthermore, in our assay, the *d14* mutant had a slightly shorter hypocotyl under mock conditions, whereas the *papp5-1 d14* double mutant did not differ from the wild-type, indicating that both genes act in separate signaling pathways (supplemental Fig. S10). Altogether, it remains unclear whether PAPP5 and KAI2 belong to the same pathway to control hypocotyl elongation. Indeed, the involvement of *PAPP5* in other light-regulated processes (57) might interfere with the final effect observed in the *papp5-1 htl-3* plants.

Several transcriptional markers of KAR and SL responses in seedlings have previously been reported, including *D14-LIKE2* (*DLK2*), *KAR-UP F-BOX1* (*KUF1*), *CHALCONE SYNTHASE* (*CHS*), *ELONGATED HYPOCOTYL 5* (*HY5*), and *SALT TOLERANCE HOMOLOG7/bzr1–1D Suppressor 1* (*STH7/BZS1*) (11, 77, 78). In view of the minor additive effect on the hypocotyl elongation in the *papp5-1 htl-3* double mutant, making the genetic experiments inconclusive, we investigated whether *PAPP5* might be involved in the impact of *rac*-GR24 on the expression of several SL/KAR marker genes in seedlings. To this end, we assessed the accumulation of several marker transcripts, of which the abundance is known to change in response to *rac*-GR24. Previously, transcript levels of *CHS*, *HY5*, *DLK2*, and *STH7* had been shown to increase after treatment with *rac*-GR24 in a MAX2-dependent manner (4, 11, 78). In contrast to prior work, *CHS* and *HY5* expression was not significantly affected by the *rac*-GR24 treatment in Col-0 seedlings (Fig. 6*C*). The *DLK2* transcript responses to *rac*-GR24 in *papp5-1* and Col-0 red-light-grown seedlings were similar, whereas the increase in the transcript level of *STH7* was significantly affected in the *papp5-1* mutant when compared with Col-0 (Fig. 6*C*). Together, these data indicate that *PAPP5* might be required for certain transcriptional responses to *rac*-GR24 in red-light-grown seedlings.

### *PAPP5* Modulates Seed Germination in Suboptimal Conditions

**Fig. 7:**
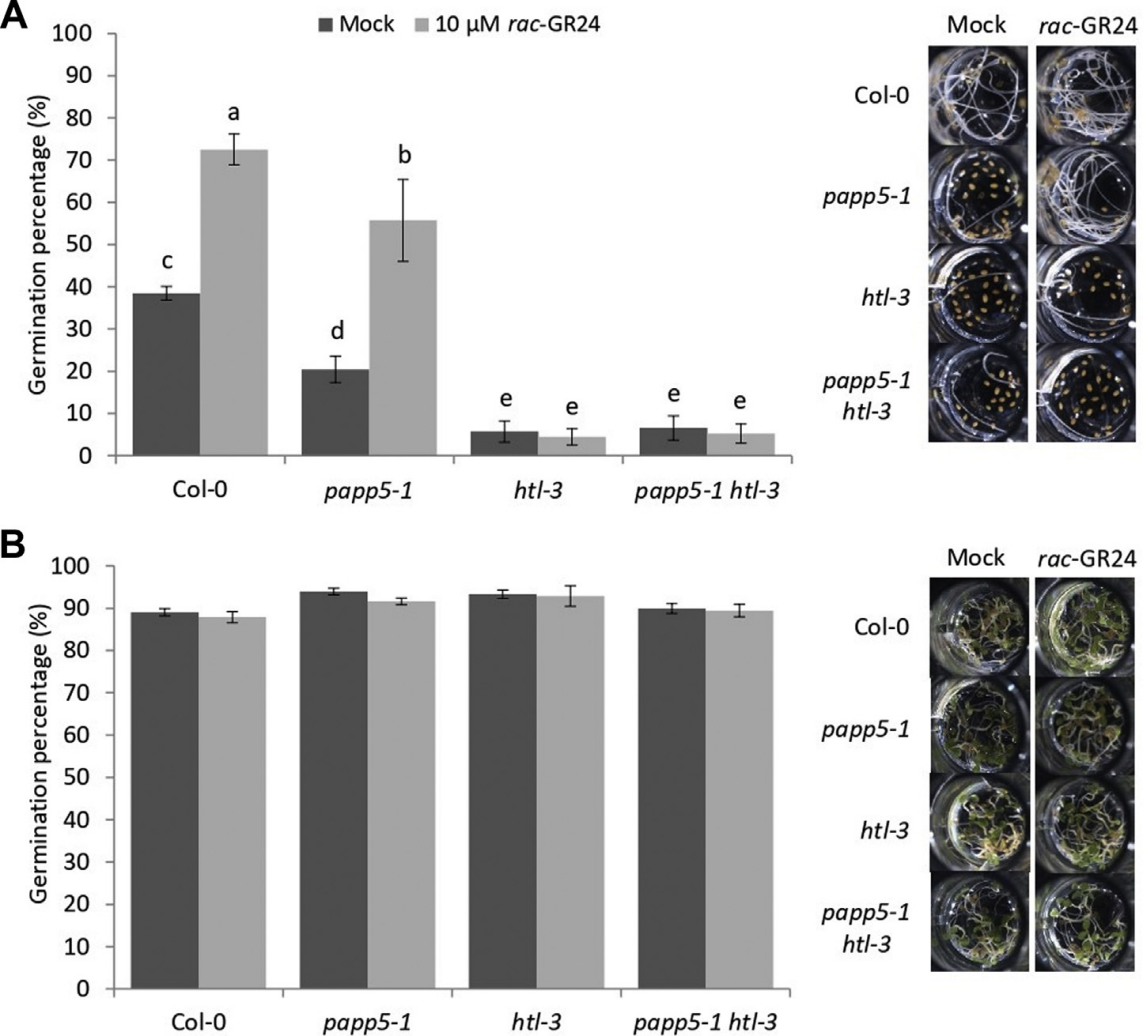
***PAPP5* regulating seed germination under suboptimal temperature conditions.** Seeds of Col-0 and the *papp5-1*, *htl-3*, and *papp5-1 htl-3* mutants were distributed in a 96-well plate containing Hepes buffer with mock (acetonitrile) or 10 μM *rac*-GR24 and placed for 4 days at 24 °C in the dark (*A*) or at 21 °C in the continuous light for the control (*B*) (*n* = 9, representing three repeats with three 96-well plates per repeat with 12 wells containing 15–40 seeds each). Values are means with error bars representing the standard error (SE). Statistical groupings (letters a-e) were determined by ANOVA mixed model with Tukey–Kramer honestly significant difference (*p* < 0.01).

Besides inhibition of the hypocotyl elongation, the KAI2 pathway also controls seed germination (11, 77). Under suboptimal temperature conditions, *Arabidopsis* Col-0 seeds respond to *rac*-GR24 by increasing the germination frequency, whereas the *max2-1* and *htl-3* mutants have a very low germination rate and are fully insensitive to the treatment (58). Under mock conditions, the germination frequency of the *papp5-1* and *papp5-3* mutants was significantly lower than that for Col-0 (Fig. 7*A*, supplemental Fig. S9*C*), suggesting that *PAPP5* might be a positive regulator of seed germination. However, for both mutants, an increase in germination rate in the presence of *rac*-GR24 was twice as high as that of Col-0 seeds (Fig. 7*A*, and supplemental Fig. S9*C*). This increase in germination frequency might indicate that *PAPP5* does not belong to the core signaling pathway but rather has a modulatory role that can be overcome by the exogenous *rac*-GR24 treatment. Under control conditions (21 °C, continuous light), all tested lines displayed a high germination frequency that was not induced by the *rac*-GR24 treatment (Fig. 7*B*).

## Discussion

In the last years, major breakthroughs have been made to unravel the signaling cascades of the structurally related SL and KAR/KL molecules. Until now, a very similar signaling mechanism has been described, consisting of an α/β-fold hydrolase receptor, D14 and KAI2, respectively, a common F-box protein MAX2, and downstream SMXL targets (4, 18, 26, 30, 33, 35, 38). However, many downstream molecular players and proteins involved in the regulation mechanisms of both pathways still await discovery.

Here, TAP was used in *Arabidopsis* cell cultures to elucidate the MAX2, D14, and KAI2 interactomes. Confirming the effectiveness of the technique, core components of the SCF^MAX2^ complex (CUL1, SKP1, ASK2, ASK4, and ASK13) were identified with the full-length MAX2 (5, 41). Furthermore, different members of the COP9 signalosome as well as eight unknown MAX2 putatively interacting proteins were detected. As none of the CSN members were retrieved with the truncated MAXΔFBOX bait, we expect that CSN plays a regulatory role, acting as a cullin deneddylase to maintain the SCF^MAX2^ activity (79), but a role in the regulation of phosphorylation and gene expression has been described as well (80, 81). In contrast to our experiments with MAX2, no putative preys were identified with D14 or KAI2 as baits. A possible explanation might be that the tag disturbed the functionality of the protein fusions, because of the only partial complementation of the corresponding mutant phenotypes. Another reason for this partial complementation might be the use of a 35S promoter that might lead to missexpression or to too high accumulation of tagged proteins. Similarly, the SMXL7 protein fusion driven by the native promoter was shown to completely restore the branching phenotype of the *smxl6 smxl7 smxl8 max2* toward the expected *max2*-like branch number, whereas the 35S promoter only partially complemented this phenotype (36).

In our TAP-MS approach, no known partners of MAX2 involved in the SL or KAR/KL pathways were copurified. One explanation could be that either the partners are low abundant or interact only transiently and, thus, are missed owing to the two-step purification procedure. Regarding the interaction with the downstream partners, prolonged treatment with *rac*-GR24 might plausibly lead to the degradation of SMXL proteins and, hence, hinder their detection (18, 35, 44).

From the newly identified interactors, we focused on PAPP5. PAPP5 is a unique serine/threonine phosphatase characterized by an amino-terminal tetratricopeptide repeat (TPR) domain that is used, instead of binding to multiple regulatory subunits, to regulate the enzyme's activity, probably the reason for its low basal phosphatase activity (82, 83). In mammalian cells, the PAPP5 homolog is described as a universal master modulator with many pleiotropic effects (82). Also, in *Arabidopsis*, PAPP5 is a versatile protein, performing multiple enzymatic activities that can be associated with its oligomeric status. In its low-molecular-weight form, PAPP5 acts as a protein phosphatase and as a foldase chaperone (enhancing protein refolding), whereas its high-molecular-weight complex is correlated with a holdase chaperone function (preventing protein aggregation) (84).

To understand the role of PAPP5 in the MAX2 signaling network, we combined biochemical studies, mutant phenotyping, and qRT-PCR analysis of SL/KAR markers. Copurification of PAPP5 with MAX2 in the TAP experiments was validated by Y2H, BiFC, and Co-IP and proved to be a direct interaction in the nucleus. Quantitative one-step APs identified PAPP5 as being significantly more associated with the KAI2 than the D14 protein complex in *Arabidopsis* cell cultures, whereas Co-IP revealed that both receptors copurify with PAPP5 when transiently expressed in *N. benthamiana*, but none of them as a direct interactor (Y2H data). Similar to the interaction between MAX2 and the receptors that is promoted by *rac*-GR24 (23, 56), the KAI2-PAPP5 association seems to depend on *rac*-GR24 in the Co-IP assay. On the contrary PAPP5 was copurified with D14 under both conditions, possibly due to endogenous SL levels in *N. benthamiana* that were sufficient to trigger this complex formation. Thus, the PAPP5 association with the receptors seems to depend on the dynamics of the D14-MAX2 and KAI2-MAX2 signaling complexes. To confirm this hypothesis, we will require interaction studies in the *max2-1* mutant background, whereas to unravel whether the complex formation is indeed dependent on *rac*-GR24, we will need SL-deficient and, still unknown, KL-deficient mutants.

Some interactors of the F-box protein MAX2 are ubiquitinated and degraded, such as the SL pathway targets, SMXL6,7,8 (32, 35, 36). Considering under which conditions PAPP5 was copurified, we tested whether it might also be a substrate of SCF^MAX2^. However, because the PAPP5 protein levels in *Arabidopsis* cell cultures remained unchanged after the *rac*-GR24 treatment, we rejected this hypothesis. Therefore, we rather expect that PAPP5 regulates the activity and/or stability of the MAX2 protein complex that might affect downstream responses. Indeed, our phosphopeptide enrichment experiment revealed that MAX2 might be a dephosphorylation target of PAPP5. The identified phosphopeptide of MAX2 has three putative phosphorylation sites, from which S116 had the highest localization probability in only one of three *papp5* samples, whereas in the AtMAX2 model structure, the S114 and S116 residues are exposed to the solvent and, therefore, possibly phosphorylated. To know the exact phosphorylation site and its biological function, the phosphomimetic and phosphodeath alleles of MAX2 in all three positions expressed in the *max2-1* mutant need to be tested for complementation. The protein sequence alignment of MAX2 orthologs showed that the detected phosphopeptide of MAX2 might also be phosphorylated in the selected species, because it contains a number of potential phosphosites. Several lines of evidence indicate that the stability or substrate interaction of E3 ubiquitin ligases can be controlled by its reversible phosphorylation (85), but the exact role of the MAX2 phosphorylation/dephosphorylation remains to be determined. Also, the phosphorylation status of MAX2 is not *rac*-GR24 dependent, indicating that there might be another stimulus that triggers this process. One possibility could be the influence of light as both proteins are implicated in light signaling (57, 72).

Reversible protein phosphorylation is essential in regulating various biological responses. Our genetic studies suggested that the *PAPP5* gene might be involved in the phenotypes controlled by the MAX2-KAI2 complex, only at the early stages of plant development. Accordingly, no difference in shoot branching and lateral root density could be observed between the *papp5* knockout mutants and the wild-type plants, whereas seed germination and seedling growth were affected. Similar to *htl-3* and *max2-1*, the hypocotyls of the *papp5-1* and *papp5-3* mutants are longer than those of Col-0, when grown under red light conditions. An additive effect on hypocotyl elongation in the *papp5-1 htl-3* double mutant suggests that both proteins might operate in separate pathways during this process. Indeed, PAPP5 had previously been found to influence phytochrome-mediated photoresponses (57). As a protein phosphatase, PAPP5 dephosphorylates the biologically active far-red light–absorbing phytochrome A, thereby increasing its ability to interact with the downstream signal transducer nucleoside-diphosphate kinase 2 (NDPK2) and, thus, to enhance the plant’s response to light. As a consequence, a defective hypocotyl growth might be caused by a disturbed phytochrome-mediated signaling cascade. Nevertheless, because the additive effect was rather small, the influence of PAPP5 on the KAI2-controlled pathway cannot be excluded. In fact, PAPP5 might be required for some transcriptional responses to *rac*-GR24 in seedlings. Under conditions identical to those used to examine the hypocotyl length, the *rac*-GR24–dependent induction of the transcript levels of *STH7*, but not of *DLK2*, was affected in the *papp5-1* mutant. *STH7* (*BZS1*) encodes a B-box zinc finger protein that has been characterized as a positive regulator in the light-signaling pathway (86). Thus, PAPP5 might provide yet another link between light and KL/KAR responses. *STH7* has been proposed to be a missing link between HY5 and *rac*-GR24 signaling during photomorphogenesis (87). However, the crosstalk between MAX2 and HY5 in the regulation of hypocotyl elongation remains unclear. Although at high concentrations, the *rac*-GR24–induced inhibition of the hypocotyl elongation depends on HY5 (88), HY5 and MAX2 have been also suggested to largely act independently from each other (89). Currently, it seems difficult to directly link the affected *STH7* expression with the elongated hypocotyl phenotype of *papp5* seedlings.

Furthermore, when the germination frequency was tested under suboptimal temperature conditions, the germination of the *papp5-1* and *papp5-3* mutants was lower than that of the wild-type plants under mock conditions, similar to the *htl-3* phenotype, hinting again at a possible involvement in the KAI2-mediated signaling. However, it is difficult to determine whether there is no additive effect in the *htl-3 papp5-1* double mutant, because *htl-3* seeds have already a very low germination frequency. The fact that *papp5* seeds are still responsive to *rac*-GR24 might be due to PAPP5 not being a main component of the signaling pathway, but to having a modulatory role. Moreover, the addition of *rac*-GR24 could mask the responses observed with the endogenous ligand levels. Taken together, based on our observations under mock conditions, PAPP5 might act as a mild positive regulator of MAX2, influencing seed germination under suboptimal conditions and seedling development.

The exact mechanism by which *PAPP5* can regulate the MAX2 pathway is still not clear. We hypothesize that, by dephosphorylation of the MAX2 protein, PAPP5 might modulate the complex activity and/or stability and facilitate the ubiquitination of SMAX1/SMXL proteins. Phosphorylation of MAX2 makes the protein less effective in the ubiquitination process, whereas dephosphorylation by PAPP5 could increase the MAX2 activity. In agreement, under mock conditions the *papp5* mutants display phenotypes similar to those of *max2* in seed germination and hypocotyl growth. Thus, a constitutively phosphorylated mutant version of MAX2 should exhibit *max2*-like phenotypes or dephosphorylation by PAPP5 might facilitate the interaction between MAX2 with its ubiquitination targets or with the D14 and KAI2 receptors. Nevertheless, the seeds and seedlings of *papp5* were still responsive to *rac*-GR24 and KAR2, possibly because the addition of the ligand might mask the responses to the physiological KL levels.

In conclusion, PAPP5 was identified as a novel MAX2 interactor. Genetic as well as biochemical analyses indicated that PAPP5 might dephosphorylate MAX2 to fine-tune seed germination under suboptimal conditions and young seedling responses. PAPP5 is seemingly a modulatory, rather than an essential, factor in the KAR/KL signaling and might link it with the light-regulatory network. Besides identification of PAPP5, we are convinced that many other interesting proteins might be hidden in our dataset, waiting for their discovery as key components in the SL and KAR/KL pathways. A detailed characterization of all members of the purified MAX2 complex will be of great value to further broaden our knowledge on the diverse MAX2 signaling networks.

## Data Availability

The mass spectrometry proteomics data have been deposited to the ProteomeXchange Consortium *via* the PRIDE (49) partner repository with the dataset identifier PXD015657. The annotated spectra for phosphopeptide enrichment and AP-MS experiments have been deposited in MS-Viewer (https://msviewer.ucsf.edu/prospector/cgi-bin/msform.cgi?form=msviewer) with the search keys: fn6jvg4j77 and q5fovjwo8j, respectively.

## Conflict of interest

Authors declare no competing interests.

## Supplementary Data

Supplemental Figures and Tables

Supplemental Excel Sheet 1
