# Supplementary material for: Unraveling the MAX2 Protein Network in Arabidopsis thaliana: Identification of the Protein Phosphatase PAPP5 as a Novel MAX2 Interactor
Source: Mol Cell Proteomics. 2021 Jan 7;20:100040. doi: 10.1074/mcp.RA119.001766 (PMC7950214; doi:10.1074/mcp.RA119.001766)
Supplement: Supplemental Figures and Tables [file mmc1.pdf]

SUPPLEMENTAL DATA

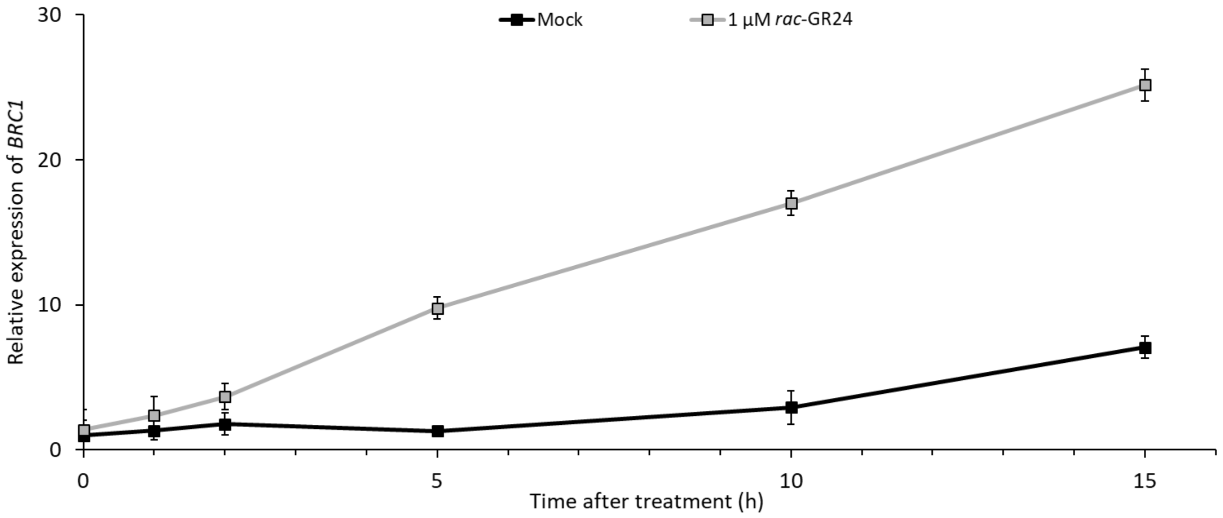

Fig. S1. **Responsivity of *Arabidopsis* cell suspension cultures to *rac*-GR24.** The expression of the *BRC1* gene was assessed by qRT-PCR in wild-type cell cultures treated with 0.01% (v/v) acetone (mock, black line) or with 1 µM *rac*-GR24 (grey line) at 0, 2, 3, 5, 10, and 15 h. The obtained expression data were normalized to the expression levels of *ACT2*. The experiment was repeated three times with comparable results and the total mean of all biological repeats is presented ± SE.

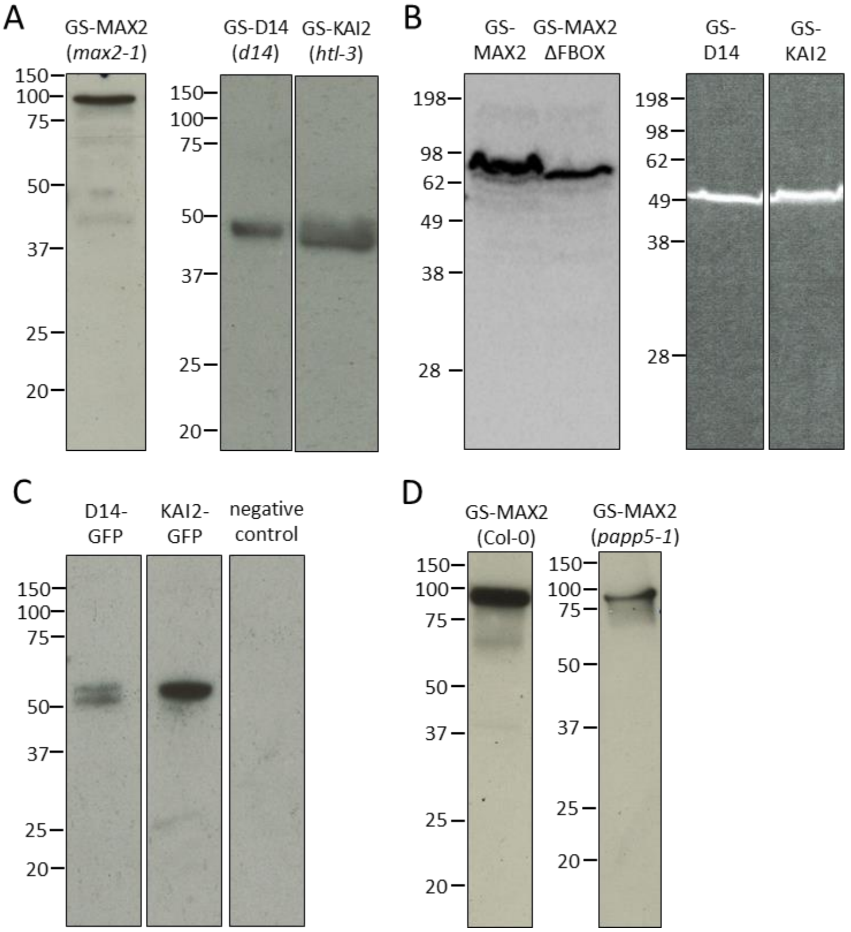

Fig. S2. **Expression analysis of bait proteins *in planta* (*A, D*) and in cell cultures (*B, C*).** Protein levels were analyzed in *Arabidopsis* cell cultures expressing *35S::GS-MAX2*, *35S::GS-MAX2ΔFBOX*, *35S::GS-D14*, and *35S::GS-KAI2* (*B*), and *35S::D14-GFP* and *35S::KAI2-GFP* (*C*), or in the transgenic lines *35S::GS-MAX2* (*max2-1*), *35S::GS-D14* (*d14-1*), and *35S::GS-KAI2* (*htl-3*) (*A*), and *35S::GS-MAX2* (Col-0) and *35S::GS-MAX2* (*papp5-1*) (*D*). Detection was done with the peroxidase–antiperoxidase (PAP) antibody against the GS tag (*A, B, E*) or against the GFP tag (*C*). For the negative control assessing the specificity of anti-GFP antibody protein extracts from *Arabidopsis* wild-type cell cultures were used. Molecular masses, 20.6 kDa for the GS-tag, 26.9 kDa for the GFP tag, 77.4 kDa for MAX2, 29.6 kDa for D14, and 29.8 kDa for KAI2.

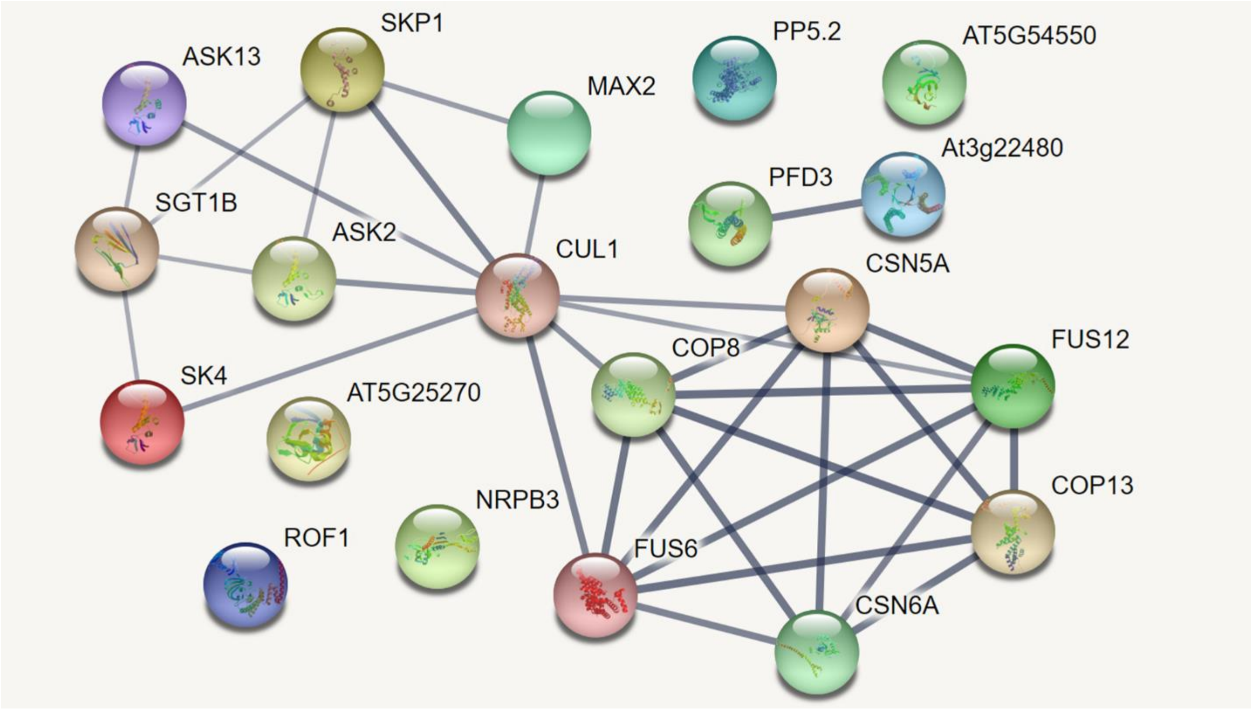

Fig. S3. **STRING network revealing connections between the prey proteins copurified with MAX2 and MAX2ΔFBOX.** All the proteins from Table 1 were analyzed and only connections based on experimental evidence are shown (confidence score > 0.400). The line thickness indicates the strength of the data support.

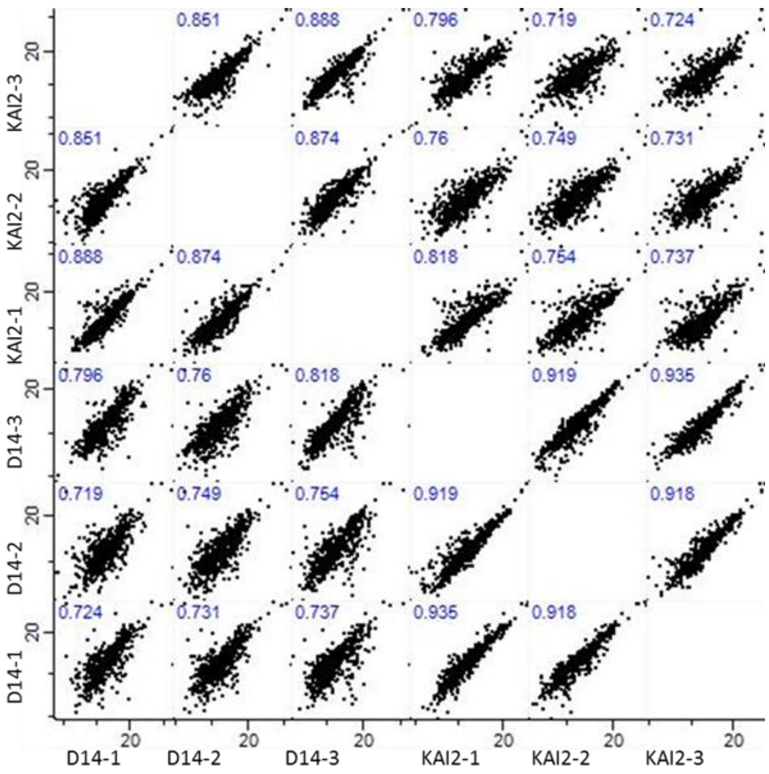

Fig. S4. **Pearson correlation coefficients for the *35S::D14-GFP* and *35S::KAI2-GFP* samples.** The matrix of 30 correlation plots reveals high correlations between LFQ intensities within replicates.

AT2G42810.1

```
1   METKNENSDV SRAEEFKSQA NEAFKGHKYS SAIDLYTKAI ELNSNNAVYW
51  ANRAFAHTKL EEYGSAIQDA SKAIEVDSRY SKGYYRRGAA YLAMGKFKDA
101 LKDFQQVKRL SPNDPDATRK LKECEKAVMK LKFEEAISVP VSERRSVAES
151 IDFHTIEVEP QYSGARIEGE EVTLDFVKTM MEDFKNQKTL HKRYAYQIVL
201 QTRQILLALP SLVDISVPHG KHITVCGDVH GQFYDLLNIF ELNGLPSEEN
251 PYLFNGDFVD RGSFSVEIIL TLFAFKCMCP SSIYLARGNH ESKSMNKIYG
301 FEGEVRSKLS EKFVDLFAEV FCYLPLAHVI NGKVFVVHGG LFSVDGVKLS
351 DIRAIDRFCE PPEEGLMCEL LWSDPQPLPG RGPSKRGVGL SFGGDVTKRF
401 LQDNNLDLLV RSHEVKDEGY EVEHDGKLIT VFSAPNYCDQ MGNKGAFIRF
451 EAPDMKPNIV TFSAVPHPDV KPMAYANNFL RMFN
```

AT2G42810.2

```
1   METKNENSDV SRAEEFKSQA NEAFKGHKYS SAIDLYTKAI ELNSNNAVYW
51  ANRAFAHTKL EEYGSAIQDA SKAIEVDSRY SKGYYRRGAA YLAMGKFKDA
101 LKDFQQVKRL SPNDPDATRK LKECEKAVMK LKFEEAISVP VSERRSVAES
151 IDFHTIGNKP RSSSMPTKTA LAAVVAAVMV VAVRGFATTE ILMVLVSVVL
201 GTFWWGSFSG KVEPQYSGAR IEGEEVTLDF VKTMMEDFKN QKTLHKRYAY
251 QIVLQTRQIL LALPSLVDIS VPHGKHITVC GDVHGQFYDL LNIFELNGLP
301 SEENPYLFNG DFVDRGSFSV EIILTLFAFK CMCPSSIYLA RGNHESKSMN
351 KIYGFEGEVR SKLSEKFVDL FAEVFCYLPL AHVINGKVFV VHGGLFSVDG
401 VKLSDIRAID RFCEPPEEGL MCELLWSDPQ PLPGRGPSKR GVGLSFGGDV
451 TKRFLQDNNL DLLVRSHEVK DEGYEVEHDG KLITVFSAPN YCDQMGNKGA
501 FIRFEAPDMK PNIVTFSAVP HPDVKPMAYA NNFLRMFN
```

Fig. S5. ***In silico* trypsin digest of both splice variants of PAPP5.** The AT2G42810.1 unique peptide SVAESIDFHTIEVEPQYSGAR, indicated in blue, was present in the peptide pool of the TAP dataset.

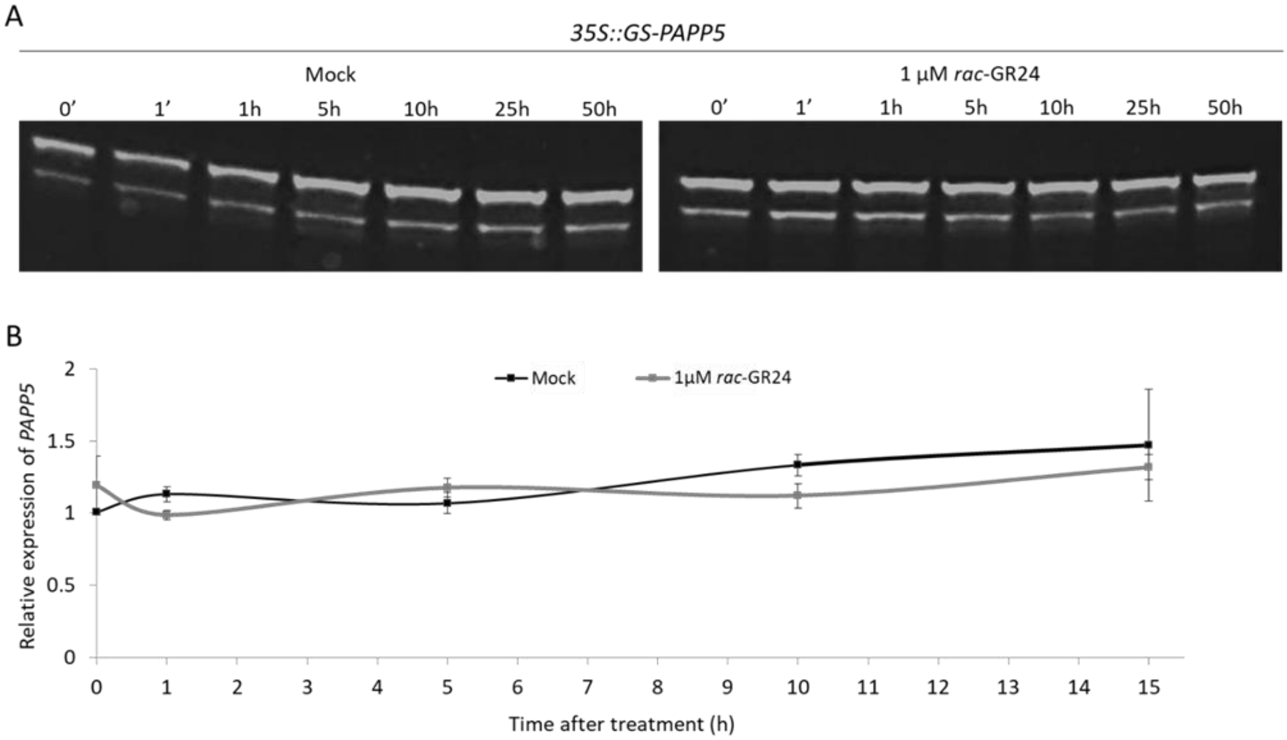

Fig. S6. **PAPP5 protein and transcript levels are unaffected by *rac*-GR24.** *A*, PAPP5 protein levels in cell cultures transformed with *35S::GS-PAPP5* at different time points upon treatment with 0.01% (v/v) acetone (mock) or with 1 µM *rac*-GR24 (min and h after treatment). Detection was done with the GS-tag antibody. Molecular masses: 20.6 kDa for the GS tag and 60.3 kDa for PAPP5. *B*, Expression of the *PAPP5* gene in wild-type cell cultures treated with 0.01% (v/v) acetone (mock, black line) or 1 µM *rac*-GR24 (grey line) at 0, 1, 5, 10, and 15 h assessed by qRT-PCR and normalized to *ACTIN2* (*ACT2*, AT3G18780). The experiment was repeated three times with comparable results and the total mean of all biological repeats is presented. Data and error bars represent means ± SE.

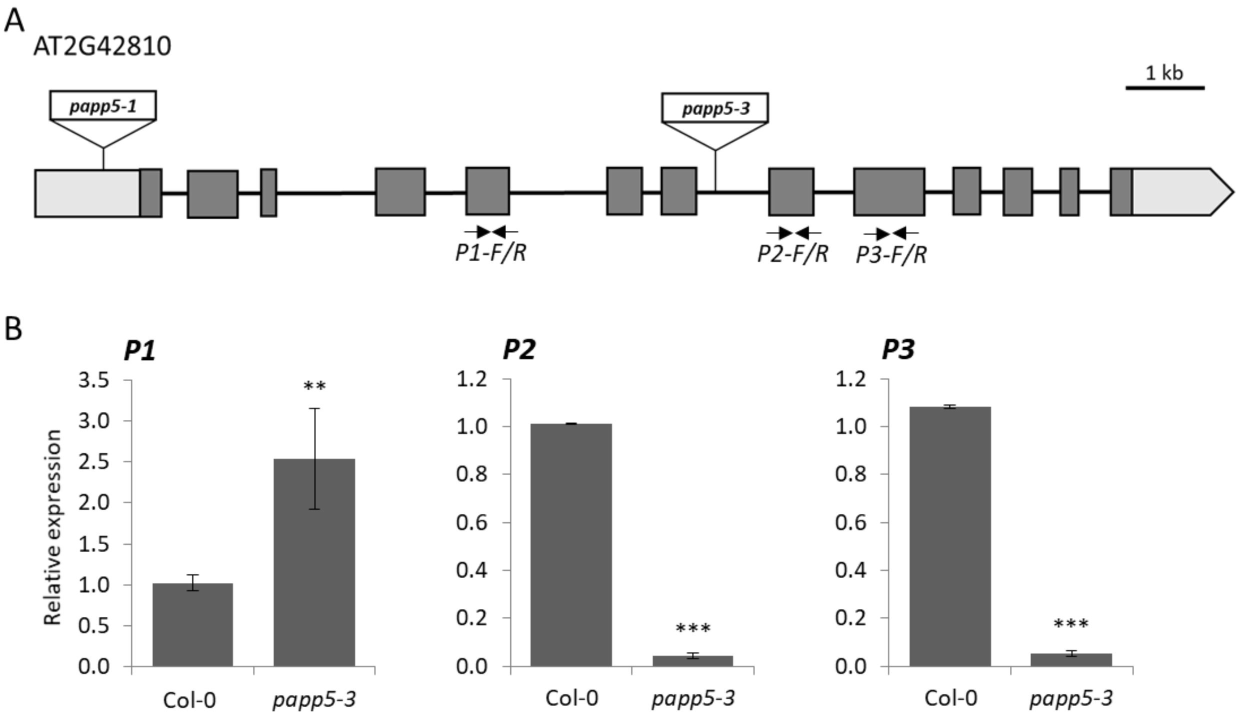

Fig. S7. **Characterization of *PAPP5* T-DNA insertion lines.** *A*, Schematic presentation of the *PAPP5* gene structures, the T-DNA insertions, and the primers used for qRT-PCR. Dark grey boxes represent exons. *B*, Transcript level of *PAPP5* in Col-0 and in the *papp5-3* mutant detected by qRT-PCR. *ACTIN2* (*ACT2*; AT3G18780) primers were used as reference gene. Error bars represent the SE, based on three independent biological repeats. Asterisks indicate statistically significant differences assessed by Student's *t* test (*** $P < 0.001$, ** $P < 0.01$).

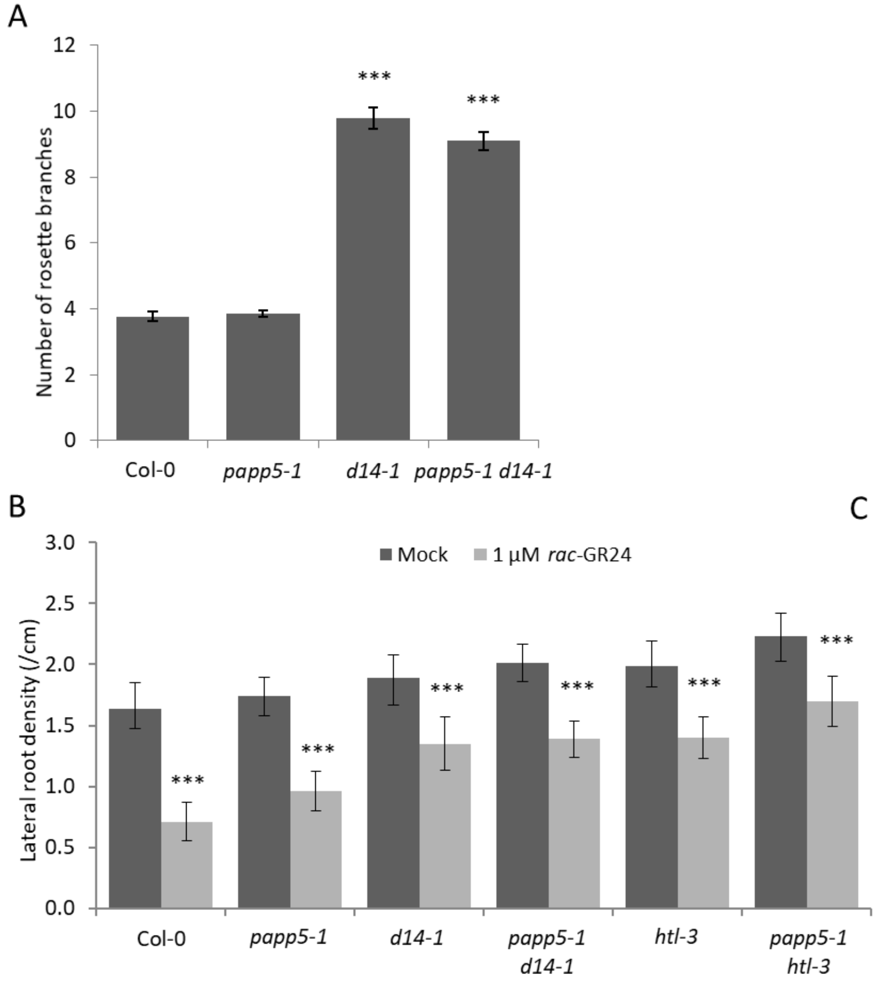

C

Genotype	% decrease	P value
Col-0	57%	-
papp5-1	45%	0.015
d14-1	33%	0.041
papp5-1 d14-1	31%	< 0.001
htl-3	30%	< 0.001
papp5-1 htl-3	37%	< 0.001

Fig. S8. ***PAPP5* is not involved in shoot branching control and lateral root development.** *A*, Rosette branches of Col-0 and the *papp5-1*, *d14-1*, and *papp5-1 d14-1* mutants counted after 6 weeks of growth (*n* = 45, representing three repeats with 15 plants per genotype per repeat**).** *B*, Effect of *rac*-GR24 on emerged lateral roots analyzed in 9-day-old seedlings (*n* = 9, representing three repeats with three plates per repeat with 30 seedlings per plate). *C,* Percentage decrease in lateral root density between mock and *rac*-GR24 treatments for each genotype. Poisson regression model was used to show statistical differences in percentage of the lateral root density decrease between the Col-0 and each mutant. Graphs present means of three biological repeats ± SE. Asterisks indicate statistically significant differences (*** $P < 0.001$ Student's *t* test [*A*], Poisson regression model [*B*]).

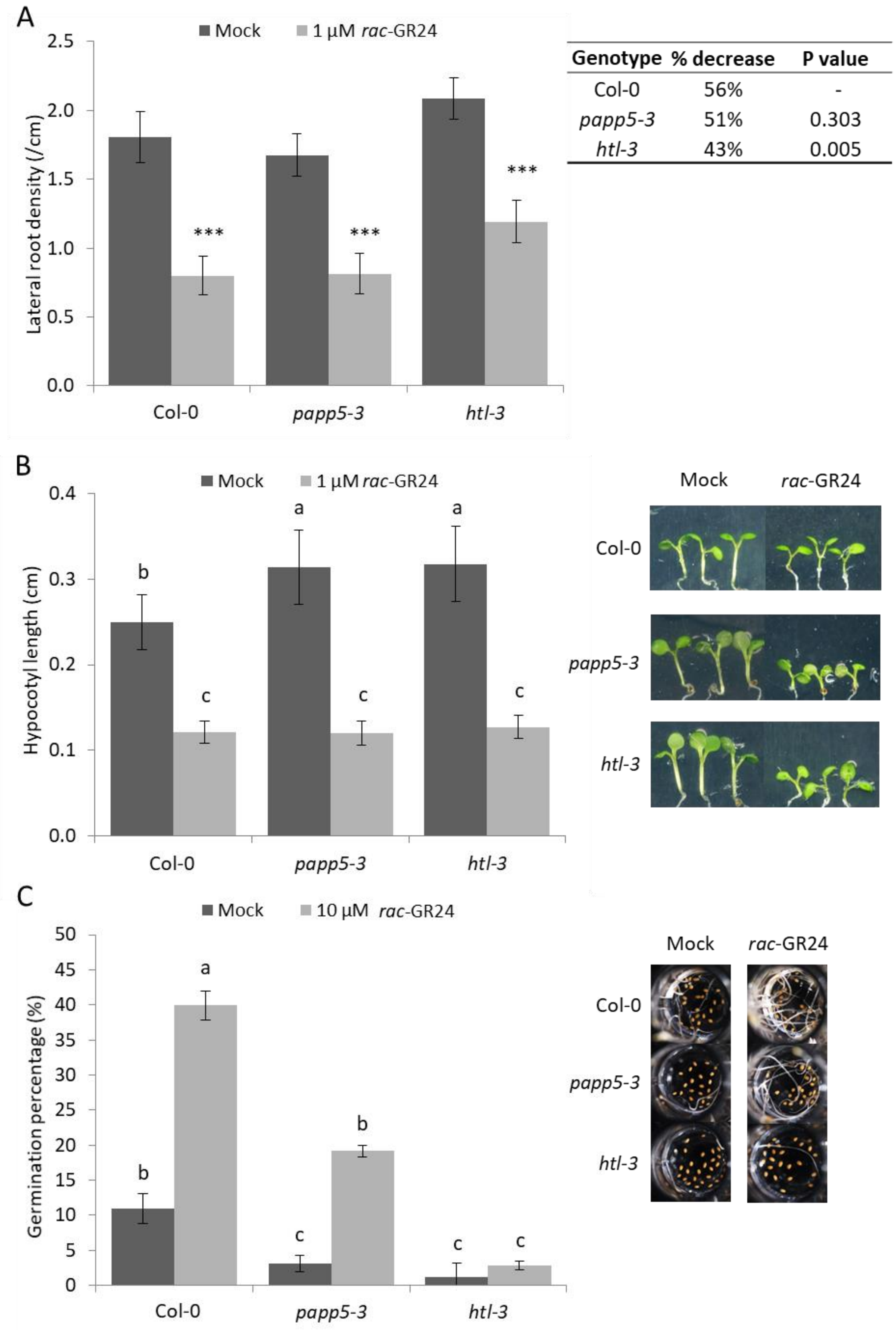

Genotype	% decrease	P value
Col-0	56%	-
papp5-3	51%	0.303
htl-3	43%	0.005

Fig. S9. **Analysis of *papp5-3* mutant phenotypes.** *A*, Effect of *rac*-GR24 on emerged lateral roots analyzed in 9-day-old seedlings (n = 9, representing three repeats with three plates per repeat and

30 seedlings per plate). The percentage decrease in lateral root density between mock and *rac*-GR24 treatments for each genotype is given in the table. *B*, Hypocotyl length measured in seedlings grown in red light for 4 days on half-strength Murashige and Skoog medium without sucrose, supplemented with 0.01% (v/v) acetone (mock) or 1 µM rac-GR24 ($n = 6$, representing three repeats with two plates per repeat and at least 35 seedlings per plate). Hypocotyls of representative Col-0, *papp5-3*, and *htl-3* mutants. *C*, Seeds of Col-0 and the *papp5-3* and *htl-3* mutants distributed in 96-well plates containing HEPES buffer with mock (acetonitrile) or 10 µM rac-GR24 and placed for 6 days at 24˚C in the dark ($n = 9$, representing three repeats with three 96-well plates per repeat with 12 wells containing 15-40 seeds each). A representative picture of one well is shown. Values are means with error bars representing the standard error (SE). Asterisks indicate statistically significant differences (*** $P < 0.001$) based on Poisson regression model (*A*). Statistical groupings were determined by ANOVA mixed model with Tukey-Kramer HSD ($P < 0.01$) (*B* and *C*).

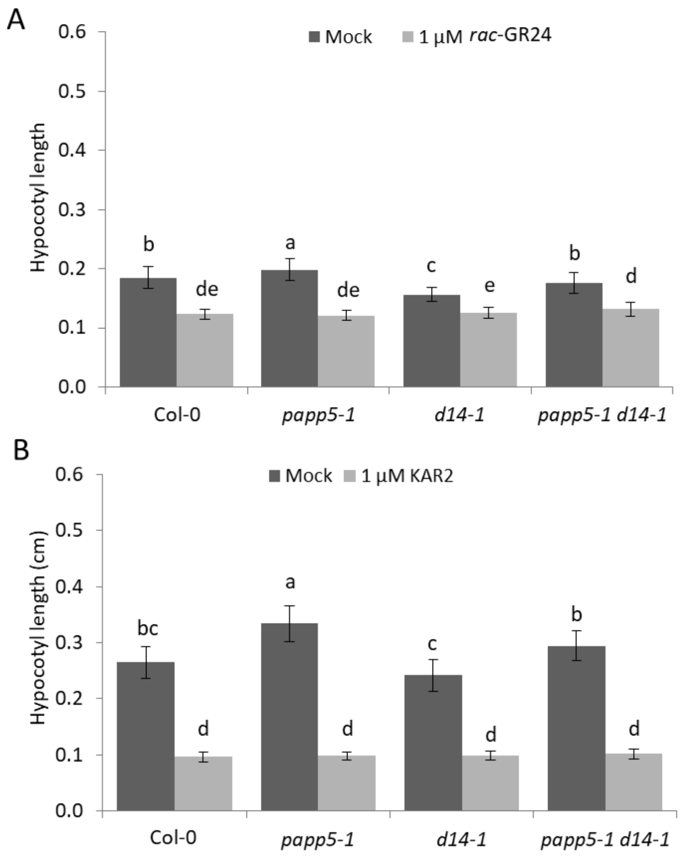

Fig. S10. **Hypocotyl responses of *papp5* and *d14* mutants to *rac*-GR24 and KAR$_2$.** The hypocotyl length measured in seedlings grown in red light for 4 days on half-strength Murashige and Skoog medium without sucrose, supplemented with (*A*) 0.01% (v/v) acetone (mock) or 1 µM rac-GR24 or (*B*) 0.01% (v/v) methanol (mock) or 1 µM KAR$_2$ ($n = 6$, representing three repeats with two plates per repeat and at least 35 seedlings per plate). Values are means with error bars representing the standard error (SE). Statistical groupings were determined by ANOVA mixed model with Tukey-Kramer HSD (*A*, $P < 0.01$ or *B*, $P < 0.05$).

Table S1. All primers used in this study.

ID		Sequence	Use
BRC1	Fw	CTTCAGCAGCGGCGATGAG	qRT-PCR
BRC1	Rev	TTCCTCTTGTTTCGGTCGTGTTAG	qRT-PCR
MAX2	Fw	GGGGACAAGTTTGTACAAAAAAGCAGGCTCAATGGCTTCCACTACTCTCTCC	Cloning
MAX2	Rev	GGGGACCACTTTGTACAAGAAAGCTGGGTATCAGTCAATGATGTTGCGGCTGTTC	Cloning
D14	Fw	GGGGACAAGTTTGTACAAAAAAGCAGGCTCAATGAGTCAACACAACATCTTAG	Cloning
D14	Rev	GGGGACCACTTTGTACAAGAAAGCTGGGTATCACCGAGGAAGAGCTCGCCG	Cloning
KAI2	Fw	GGGGACAAGTTTGTACAAAAAAGCAGGCTCCACCATGGGTGTGGTAGAAGAAGCTC	Cloning
KAI2	Rev	GGGGACCACTTTGTACAAGAAAGCTGGGTCTCACATAGCAATGTCATTACGAAT	Cloning
MAX2ΔFBOX	Fw	GGGGACAAGTTTGTACAAAAAAGCAGGCTCAATGGCTCGTGGCAACGCTCGTGATC	Mutagenesis
PAPP5	Fw	GGGGACAAGTTTGTACAAAAAAGCAGGCTCAATGGAGACCAAGAATGAGAATTCTG	Cloning
PAPP5	Rev	GGGGACCACTTTGTACAAGAAAGCTGGGTATTAGTTGAACATCCTGAGAAAGTTG	Cloning
pMAX2	Fw	GGGGACAACTTTGTATAGAAAAGTTGCTAACGGTTGAATTGTATACATGG	Cloning
pMAX2	Rev	GGGGACTGCTTTTTTGTACAAACTTGCGAGAAGCGGCAAATCTACAAG	Cloning
PAPP5	Fw	TGTCTCACTCCTCGTCAACCT	qRT-PCR
PAPP5	Rev	TTGTGGCTTCACCGGATAAT	qRT-PCR
DLK2	Fw	GCTGCTTCTCCAAGGTATATAA	qRT-PCR
DLK2	Rev	GAAATCAACCGCCCAAGCT	qRT-PCR
STH7	Fw	CATCTCCCGTTCTCTCTCACTTCT	qRT-PCR
STH7	Rev	CATTCTCTGCATAGTATTCCTCTGCT	qRT-PCR
HY5	Fw	AGAGGTCATCAAGCTCTGCTCCAC	qRT-PCR
HY5	Rev	TCCGACAGCTTCTCCTCCAAACTC	qRT-PCR
CHS	Fw	GGCTATTGGCACTGCTAACCCTGAG	qRT-PCR
CHS	Rev	GTGACGTTTCCGAATTGTCGACTTG	qRT-PCR
ACT2	Fw	GGCTCCTCTTAACCCAAAGGC	qRT-PCR
ACT2	Rev	CACACCATCACCAGAATCCAGC	qRT-PCR
P1	Fw	TGCCCACCAAAACGGCTTTA	qRT-PCR
P1	Rev	TCAGTTGTGGCAAATCCCCG	qRT-PCR
P2	Fw	CCCATACCTATTTAATGGCGAC	qRT-PCR
P2	Rev	CGAACCTCACCCTCAAAAC	qRT-PCR
P3	Fw	CTGTGAGCCACCAGAGGAAG	qRT-PCR
P3	Rev	TTTGGCGCAGAGAAGACAGT	qRT-PCR

Table S2. Overview of proteins significantly more associated with KAI2 or D14 in the GFP trap experiment.

AGI	Protein	-LOG(P-value)	Difference (D14/KAI2)
**KAI2**			
AT1G02930	GSTF6		
AT1G02920	GSTF7		
AT4G02520	GST2		
AT2G02930	GSTF3	3.34	6.42
AT1G29250.1			
AT2G34160.1	Alba DNA/RNA-binding protein	2.45	3.28
AT1G51410.1	NAD(P)-binding Rossmann-fold superfamily protein	2.50	2.91
AT2G42810	PAPP5	2.78	2.88
AT1G18080.1	ATARCA	2.18	2.83
AT1G15340	MBD10	2.49	2.72
AT1G48630.1	RACK1B_AT		
AT3G18130.1	RACK1C_AT	2.04	2.51
AT1G22450.1	COX6B	2.04	2.46
AT1G78300.1	GRF2	1.58	2.25
AT3G20050.1	TCP-1	2.12	2.11
AT2G39020.1	Acyl-CoA N-acyltransferases (NAT) superfamily protein	1.83	1.97
AT2G42680.1	MBF1A		
AT3G58680.1	MBF1B	1.70	1.80
AT3G25230	ROF1	2.53	1.74
AT2G42590	GRF9	2.10	1.71
AT1G07750.1			
AT2G28680.1	RmlC-like cupins superfamily protein	1.94	1.69
AT3G16640	TCTP	2.09	1.67
AT2G45290.1	Transketolase	1.68	1.58
AT3G11830	TCP-1/cpn60 chaperonin family protein	2.11	1.57
AT2G04030	AtHsp90.5	2.36	1.56
AT1G23100.1	GroES-like family protein	2.72	1.22
AT2G45300.1	RNA 3'-terminal phosphate cyclase/enolpyruvate transferase,		
AT1G48860	alpha/beta	1.91	1.21
AT3G17820	ATGSKB6	1.87	1.16
AT1G53240.1	mMDH1		
AT3G15020.1	mMDH2	2.28	1.13
AT2G28000	CPN60A	1.94	1.02
AT1G80270	PPR596	2.84	0.63
**D14**			
AT3G05060.1	NOP56-like pre RNA processing ribonucleoprotein	2.14	0.96
AT3G27240.1	Cytochrome C1 family	2.06	0.97
AT3G04400			
AT2G33370.1	Ribosomal protein L14p/L23e family protein		
AT1G04480.1		2.06	0.97
AT1G55900	TIM50	1.92	1.06
AT2G21390.1	Coatomer, alpha subunit	2.52	1.08
AT4G09800.1	RPS18C		
AT1G34030.1	Ribosomal protein S13/S18 family		
AT1G22780.1	RPS18A	2.03	1.33
AT2G19730	Ribosomal L28e protein family	2.17	1.35
AT1G70770	Protein of unknown function DUF2359	1.99	1.36

AT2G45710.1<br>AT3G61111.1<br>AT5G47930.1<br>AT3G61110.1	Zinc-binding ribosomal protein family protein	2.39	1.49
AT2G35900.1	Unknown protein	1.65	1.87
AT1G43890.3	ATRAB18	3.00	1.89
AT3G12580.1	HSP70	1.88	1.98
AT1G68680.1	Unknown protein	1.62	2.04
AT3G44110	ATJ3	2.50	2.27
AT5G67380<br>AT3G50000.1<br>AT2G23070.1<br>AT2G23080.2	 CKA1<br>CKA2<br>Protein kinase superfamily protein	2.12	2.48
AT5G22880.1<br>AT3G46030.1<br>AT1G07790.1<br>AT5G59910.1<br>AT3G45980.1<br>AT2G28720.1<br>AT5G02570.1<br>AT2G37470.1<br>AT3G09480.1<br>AT3G53650.1	 <br>H2B<br>HTB11<br>HTB1<br>HTB4<br>HTB9<br>Histone superfamily protein	1.91	2.51
AT1G56110.1<br>AT3G12860.1	NOP56 | NOP56-like pre RNA processing ribonucleoprotein	1.58	2.81
AT2G28740.1<br>AT3G45930.1<br>AT5G59970.1<br>AT5G59690.1<br>AT3G53730.1<br>AT3G46320.1<br>AT1G07820<br>AT1G07660.1	 <br>HIS4<br>Histone superfamily protein	2.65	2.93
AT2G06850.1	EXGT-A1	1.90	3.47
AT5G54640.1<br>AT4G27230<br>AT1G51060.1<br>AT3G20670.1<br>AT1G54690.1<br>AT1G08880.1	HTA1<br>HTA2<br>HTA10<br>HTA13<br>HTA3<br>HTA5	2.02	4.16

Proteins found significantly different between D14 and KAI2 are highlighted. Student's *t* test results from the Perseus software on a set of LFQ values from three independent single-step affinity purification experiments done in cell cultures expressing *35S::D14-GFP* and *35S::KAI2-GFP* as bait, treated with 1 µM *rac*-GR24.
